# *EGb* in the Treatment for Patients with VCI: A Systematic Review and Meta-Analysis

**DOI:** 10.1155/2021/8787684

**Published:** 2021-08-27

**Authors:** Min Zhan, Linjuan Sun, Jianxun Liu, Zixiu Zeng, Wei Shen, Huimin Li, Ying Wang, Fuhua Han, Jingzi Shi, Xinyun Zeng, Xiyue Lu, Yunling Zhang, Xing Liao

**Affiliations:** ^1^Xiyuan Hospital, Chinese Academy of Traditional Chinese Medicine, Beijing 100091, China; ^2^Center for Evidence-Based Chinese Medicine, Institute of Basic Research in Clinical Medicine, China Academy of Chinese Medical Sciences, China; ^3^Graduate School, Chinese Academy of Traditional Chinese Medicine, Beijing 100700, China; ^4^Graduate School, Beijing University of Traditional Chinese Medicine, Beijing 100029, China

## Abstract

**Background:**

*Ginkgo biloba* extract (EGb) is widely used to treat impairments in memory, cognition, activities of daily living, inflammation, edema, stroke, Alzheimer's dementia, and aging.

**Aim:**

We aimed to evaluate the safety and efficacy of EGb in treating vascular cognitive impairment (VCI).

**Methods:**

The systematic review was performed using the latest guidelines. We searched for EGb-related trials up to March 1, 2021, in four Chinese databases, three English databases, and clinical trial registry platforms. Randomized controlled trials (RCTs) were included if the study enrolled participants with VCI. Two reviewers independently extracted the data and critically appraised the study quality. Heterogeneity was quantified with *I*^2^. Both sensitivity and subgroup analyses were used to identify the sources of heterogeneity. Publication bias was assessed with funnel plots. We used the Grading of Recommendations Assessment, Development, and Evaluation (GRADE) approach to rate the evidence quality. Outcomes included assessments using the Activities of Daily Living (ADL), Montreal Cognitive Assessment (MoCA), Mini-Mental State Examination (MMSE), Hasegawa Dementia Scale (HDS), Barthel Index (BI), Functional Activity Questionnaire (FAQ), and adverse events.

**Results:**

In this study, a total of 2019 patients in 23 RCTs were included. EGb appeared to be more effective than control conditions as assessed by the results of cognitive function evaluation, including MMSE (MD_MMSE,EGb vs.blank_ = 3.04, 95% CI: 0.10-5.98; MD_MMSE,EGb vs.drugs for VCI_ = 2.70, 95% CI: 1.39-4.01; MD_MMSE,EGb+drugs for VCI vs.blank_ = 5.90, 95% CI: 4.21-7.59; and MD_MMSE,EGb+drugs for VCI vs.drugs for VCI_ = 3.14, 95% CI: 2.14-4.15), MoCA (MD_MoCA,EGb vs.blank_ = 5.30, 95% CI: 2.15-8.46; MD_MoCA,EGb+drugs for VCI vs.blank_ = 2.66, 95% CI: 1.82-3.50; and MD_MoCA,EGb+drugs for VCI vs.drugs for VCI_ = 2.56, 95% CI: 1.85-3.27), HDS (MD_HDS,EGb vs.blank_ = 6.50; 95% CI: 4.86-8.14; MD_HDS,EGb+drugs for VCI vs.drugs for VCI_ = 3.60, 95% CI: 2.50-4.70), ADL (MD_ADL,EGb vs.blank_ = 7.20, 95% CI: 3.28-11.12; MD_ADL,EGb+drugs for VCI vs.blank_ = 10.00, 95% CI: 7.51-12.49; and MD_ADL,EGb+drugs for VCI vs.drugs for VCI_ = 9.20, 95% CI: 7.26-11.14), BI (MD_BI,EGb+drugs for VCI vs.drugs for VCI_ = 5.71, 95% CI: 2.99-8.43; MD_FAQ,EGb vs.drugs for VCI_ = −1.43, 95% CI: -2.78 to 0.08), and FAQ (MD_FAQ,EGb+drugs for VCI vs.drugs for VCI_ = −2.17, 95% CI: -4.13 to 0.21). Evidence of certainty ranged from medium certainty to very low certainty.

**Conclusion:**

This meta-analysis showed that EGb may be an effective and safe treatment in improving MMSE, MOCA, ADL, and BI for VCI patients within three months of diagnosis. However, given the quality of the included RCTs, more preregistered trials are needed that explicitly examine the efficacy of EGb. This systematic review has been registered on PROSPERO, with the registration number CRD42021232967.

## 1. Introduction

Vascular cognitive impairment (VCI) may occur as a consequence of cardiovascular disease (CVD) and covers a broad spectrum of cognitive dysfunction, ranging from subjective cognitive decline and mild cognitive impairment to dementia [1, 2]. There is little consistency in the overall incidence of VCI, possibly because of different settings and designs, as well as neuroimaging accessibility [3, 4]. VCI is a clinical syndrome that occurs as a result of many different vascular pathologies [5, 6]. As a general statement, any disease process causing cerebral ischemia or hemorrhage can cause VCI. Therefore, VCI may become the silent epidemic of the 21st century [7]. The Guidelines from the Vascular Impairment of Cognitive Classification Consensus Study (VICCCS), International Society for Vascular Behavioural and Cognitive Disorders (VASCOG), and the Diagnostic and Statistical Manual of Mental Disorders, Fifth Edition (DSM-5) have divided VCI into mild VCI and major VCI according to the severity of cognitive impairment. There are four subtypes of major VCI, including poststroke dementia (PSD), pubcortical ischemic vascular dementia (SIVaD), multi-infarct dementia (MID), and mixed dementias (MixD) [8, 9]. The clinical features of VCI are variable, depending on the type, extent, and location of the underlying cerebrovascular pathology, and include memory problems, mental slowness, and problems with executive functions. VCI is the second most common cause of dementia, accounting for 15% of dementia cases [10], with a higher prevalence of vascular dementia (VaD) in the elderly in Asia [11]. With the rise in life expectancy over the past century, the number of people affected by dementia is likely to rise. Patients with VaD have a higher level of disability and higher rates of cerebrovascular diseases, congestive heart failure, hemiplegia, paraplegia, myocardial infarction, and a higher relative risk of death compared to Alzheimer disease (AD), thus increasing both the complexity and costs of management of the disease [12, 13]. VCI represents a global problem and poses a substantial economic cost to public health systems and society in Asia now and in the near future [14]. Studies have found that VCI is common and suggest it to be an important target for treatment because it may be preventable [15]. Interventions against potentially modifiable risk factors associated with VCI [16], such as controlling diabetes and hypertension and avoiding midlife obesity, among others, have been proposed as ways of reducing dementia [17].

Extracts of the leaves of the maidenhair tree, *Ginkgo biloba*, have long been used for treating various disorders and are one of the most widely used plant-based products. Standardized extracts are prescribed for the treatment of various disorders, including cognitive dysfunction, headache, tinnitus, vertigo, inattention, mood disturbances, cardiovascular disease [18], coronary heart disease [19, 20], and age-related macular degeneration [21]. *Ginkgo biloba* is mainly used in the treatment of cerebral dysfunction. The consensus of the Asian Clinical Expert Group on Neurocognitive Disorders in 2019 recommended EGb as an important part of the clinical treatment of neurodegenerative diseases, such as AD, which has received widespread attention [22, 23]. The active components of *Ginkgo biloba* consist of flavonoids, terpenoids, ginkgolides, and bilobalide. *Ginkgo biloba* has been demonstrated to have antioxidative activity and has been shown to restore impaired mitochondrial function, thereby improving the neuronal energy supply, as well as improving compromised hippocampal neurogenesis and neuroplasticity [24], inhibiting the aggregation and toxicity of the amyloid *β*-peptide [25], decreasing blood viscosity, enhancing microperfusion [26], and increasing dopamine levels in the rat prefrontal cortex thus enhancing working memory and executive control [27]. Current studies have shown that EGb can influence the PI3K/Akt, CREB, and RSK1/GSK-3*β* signaling pathways to play a neuroprotective role [28]. Two well-defined extracts, EGb 761 and Kaveri (LI 1370), are produced from the ground leaves. In Germany, EGb 761 is one of the top five prescription medicines, while it is marketed as a food supplement and available without prescription in the UK, Canada, and the USA [28]. *Ginkgo biloba* has been the subject of many research reports and has been investigated in numerous clinical trials. Many systematic reviews covering different aspects of *Ginkgo biloba* have been published [21, 28, 29].

Despite the number of clinical trials conducted to assess its potential properties [30, 31] and the publication of several reviews documenting its efficacy in the prevention of cognitive decline and for treating cognitive impairment and dementia [32], there is still no compelling evidence on the efficacy of EGb for VCI. Therefore, we conducted a systematic review to evaluate the efficacy and safety of EGb for VCI.

## 2. Materials and Methods

This meta-analysis is reported in accordance with the Preferred Reporting Items for Systematic Reviews and Meta-Analyses (PRISMA) statement [33], displayed in Appendix 1.

### 2.1. Data Sources and Searches

Relevant studies were identified by searching seven databases and two trial registration platforms from their inception to March 1, 2021. The databases included the Chinese Biological Medical Literature Database, Chinese Wanfang data, Chinese VIP information, Chinese National Knowledge Infrastructure, PubMed, EMBASE, and the Cochrane library. The trial registration platforms were the China Clinical Trial Registration Center (ChiCTR) and ClinicalTrials.gov. We also checked the reference lists of all retrieved articles and relevant review articles to identify additional studies. We only paid attention to studies published in English and Chinese. Two individual reviewers (MZ and ZXZ) screened the titles and abstracts to select relevant studies, and duplicate studies were removed after screening each article's abstract and title. Subsequently, the eligibility criteria were used to review full-text manuscripts for available data. Any disagreements were settled via a consensus with a third researcher (XL). The detailed search strategies are shown in Appendix 2.

### 2.2. Study Selection

This systematic review employed the PICOS strategy, an abbreviation of patient, intervention, comparison, and outcome which was used for all steps in the current systematic review. Firstly, randomized controlled trials (RCTs) that examined the efficacy of EGb for VCI were included. Secondly, we used VCI as the umbrella term encompassing vascular dementia and other cognitive syndromes with a presumed vascular basis (including mild VCI and all subdivisions of major VCI). Thirdly, we included trials with the intervention of EGb (tablets) alone or combined with a drug for VCI (hereafter referred to as DV and mainly including drugs to promote microcirculation and improve cognition, such as donepezil, nimodipine, huperzine, oxiracetam, piracetam, and butylphthalide, among others). There are three common forms of oral *Ginkgo* leaf products, namely, tablets, capsules, and soft capsules. It has been found in clinical practice that patients prefer tablets than the other two forms. Fourthly, the control therapy could be any kind of DV, blank, or placebo. Normally, patients would be prescribed some basic supporting treatments, including symptomatic treatment for hyperglycemia, hyperlipidemia, and hypertension. The blank or placebo would then be added to the basic supporting treatments. Fifthly, the outcomes covered measurements of cognitive function, including the Mini-Mental State Examination (MMSE), Montreal Cognitive Assessment (MoCA), and Hasegawa Dementia Scale (HDS), as well as the evaluation of daily activities, including the Activities of Daily Living (ADL), Barthel Index (BI), and Functional Activity Questionnaire (FAQ) assessments, and safety was assessed by the occurrence of adverse reactions/events. We excluded studies if they included any of the following: (1) no available full text; (2) non-RCTs (i.e., editorials, commentaries, and letters to the editor); (3) studies with faulty data; and (4) duplicate studies.

### 2.3. Data Extraction and Quality Assessment

All related records from databases and platforms were imported into the literature management software NoteExpress 3.2.0. The eligible studies were independently screened and selected by two reviewers (MZ and ZXZ). Then, the key information was extracted from the included studies using standardized data extraction forms, including information on the authors and study design, participant characteristics, details of the intervention and control groups, and the outcomes. The risk of bias of the included trials was evaluated by two reviewers (MZ and ZXZ) according to the Cochrane risk of bias assessment tool (Cochrane Reviewers Handbook version 6.1) [34]. Seven items were evaluated, including (1) random sequence generation, (2) allocation hiding, (3) blind setting (researchers, subjects), (4) blind evaluation of study outcomes, (5) data integrity of outcomes, (6) selective reporting of research results, and (7) Other sources of bias (such as potential bias related to special research design, baseline imbalance, and suspected fraud). Each item was evaluated as being “low risk,” “unclear,” or “high risk” and was independently completed by two evaluators (MZ and ZXZ). Disagreements during the study screening, data extraction, and quality appraising were resolved by consulting a third reviewer (XL).

### 2.4. Data Synthesis and Analysis

The GRADE (the Grading of Recommendations Assessment, Development, and Evaluation) system [35] was used to rate the quality of a body of evidence across outcomes. GRADE has four levels of evidence, also known as certainty in evidence or quality of evidence: very low, low, moderate, and high. We assessed the five aspects for each outcome (risk of bias, inconsistency, imprecision, indirectness, and publication bias) to evaluate the quality of the body of evidence as it related to the studies that contributed data to the meta-analyses. We created a “Summary of findings” table to summarize the effects of interventions on key outcomes, including MMSE, MoCA, HDS, ADL, BI, FAQ, and serious adverse events. We used GRADEpro GDT [36] to create the “Summary of findings.” Explanations for downgrading the quality of the evidence were listed in the footnotes.

Data analysis was performed by Review Manager 5.3 software [37] provided by the Cochrane Collaboration. We calculated the weighted mean difference (WMD) with 95% confidence interval (CI) for continuous data, and the risk ratio (RR) with 95%CI was computed for the dichotomous data. For continuous data, if the outcome was measured on different assessment scales (such as pain), we calculated the standardized mean differences (SMDs) with 95% CIs. Before we performed the meta-analysis, the clinical heterogeneity and methodological heterogeneity were assessed. If there was no clinical or methodological heterogeneity, the chi-square test with a significance level at *P* < 0.1 and the *I*^2^ statistic were used to quantify possible heterogeneity. If *P* ≥ 0.10 and *I*^2^ < 50%, there was no statistical heterogeneity between the studies in the meta-analysis and the fixed effects model was used for analysis. If *P* < 0.10 and *I*^2^ > 50%, this was considered to represent substantial heterogeneity between studies in the meta-analysis, and the random effects model was used to pool the data. Sensitivity or subgroup analyses were performed to determine the reasons for heterogeneity and whether the random effects model could be used for analysis or not. Descriptive analysis was used if the clinical heterogeneity was too large, or there were insufficient reports to perform a meta-analysis. All analyses were two-tailed, with alpha set at 0.05, except for heterogeneity. Publication bias was assessed using funnel plots for more than 10 studies with a particular outcome.

## 3. Results

### 3.1. Literature Search and Trial Selection

**Figure 1:**
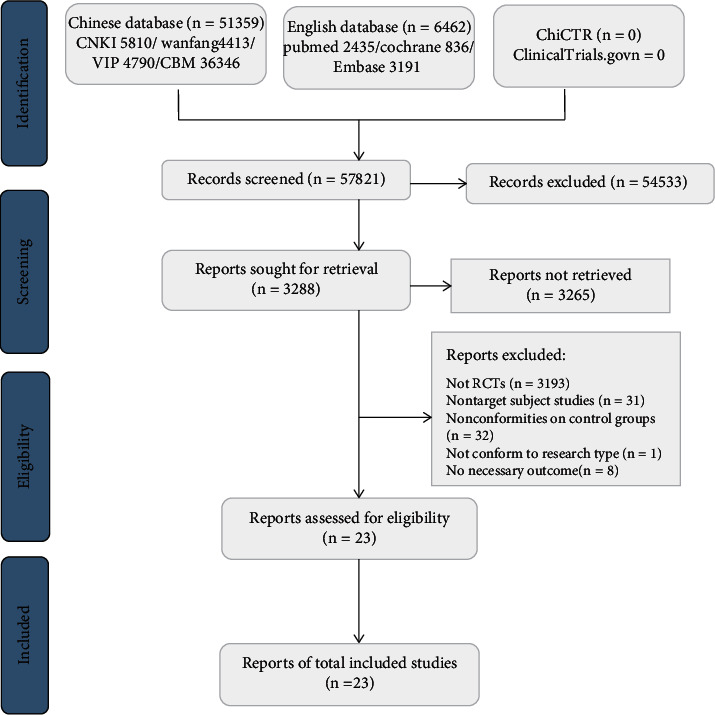
Flow chart of the study selection process.

The original search of the above nine databases yielded 57,821 electronic records, including 6462 records in English and 51,359 records in Chinese. After screening the titles and abstracts, the full text of 3288 articles was reviewed. Ultimately, 23 eligible studies were selected for the present review [38–60] and were included in the qualitative and quantitative synthesis. The screening process is summarized in a flow diagram shown in Figure 1. A self-evaluation according to the PRISMA checklist is shown in Appendix 1.

### 3.2. Description of the Included Trials

In this study, 23 RCTs on the use of EGb in the treatment of VCI were included. All trials were performed in mainland China and published in Chinese. A total of 2019 patients were included, including 1012 patients in the experimental group and 1007 patients in the control group. The patients' ages ranged from 49 to 83 years. There were no significant differences in sex, age, course of disease, or condition of the subjects between the study groups, with comparable baselines. Among the 23 RCTs, four assessed poststroke cognitive impairment (PSCI) [44, 50, 51, 56]; three studied vascular cognitive impairment, no dementia (VCIND) [47, 53, 59]; one did not mention sepcial subtype [42]; the remaining 15 reported on vascular dementia (VD) [38–41, 43, 45, 46, 48, 49, 52, 54, 55, 57, 58, 60]. Eleven studies [39, 42–45, 47, 48, 52, 53, 55, 56] used the diagnostic criteria of the Neurology Society of Chinese Medical Association, seven studies [38, 40, 41, 46, 49, 54, 57] used the American Psychiatric Association criteria, and the remaining five studies [50, 51, 58–60] were unclear. Sixteen RCTs [38, 40–47, 49–54, 60] used EGb in combination with DV (eight trials [42–45, 50–52, 60] with donepezil, three trials [38, 47, 53] with nimodipine, five trials [40, 41, 46, 49, 54] with huperzine, oxiracetam, piracetam, butylphthalide, ergoloid, and XueSaiTong) as the treatment group versus DV as the control group. Two RCTs [48, 57] used EGb as the monotherapy in the treatment group versus DV alone in the control group. Three RCTs [39, 55, 59] used EGb as the monotherapy in the treatment group versus a blank group. The duration of studies lasted from two to six months. As the outcome measurements, sixteen studies [38–40, 43, 44, 46, 48–52, 54–57, 60] used MMSE, nine studies [39, 42, 45–47, 50, 53, 58, 59] used MoCA, six studies [38–41, 45, 56] used ADL, three studies [44, 51, 55] used HDS, four studies [43, 46, 54, 60] used BI, and three studies [48, 49, 57] used FAQ. The total clinical efficacy rate was observed in 11 studies [41, 43–45, 48–50, 54, 55, 57, 60]. Adverse effects were reported in 15 studies [38–40, 43–45, 48–52, 54, 56, 57, 60]. The characteristics of the 23 trials are summarized in Table 1.

### 3.3. Risk of Bias Assessment of the Included Studies

**Figure 2:**
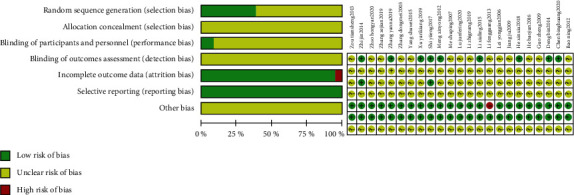
Methodological quality graph: review authors' judgments about each methodological quality item presented as percentages across all included studies and each item for each included study.

All 23 trials claimed randomization, however, none of them described the allocation of concealment methods, and none used placebo controls or registered their protocols. The method of random sequence generation was described in eight trials as a random number table [41–44, 47, 50, 53, 54]; others did not report specific methods. Only one trial used a single-blind method for patients [42]; one trial used a double-blind method for both patients and researchers [46]. Adverse events were reporeted in 15 trials; only one trial reported dropout [38]. The selective reporting assessments of all RCTs were defined as “low” for their clear inclusion and exclusion criteria. The results of the risk of bias assessment of the included studies are shown in Figure 2.

### 3.4. Meta-Analysis

#### 3.4.1. Analysis of MMSE

**Figure 3:**
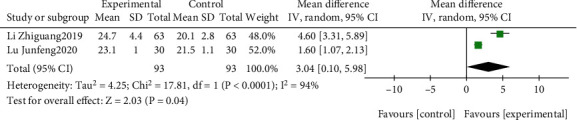
Forest plot of comparison: EGb versus blank group on MMSE levels.

The effect of EGb compared to the blank on MMSE is summarized in Figure 3. We used random effects models for pooling the effect estimates in two studies (*n* = 186 patients) [39, 55]. There was a significant difference in favor of EGb for improving MMSE (MD: 3.04; 95% CI: 0.10-5.98; *P* = 0.04). However, the heterogeneity was substantial (*I*^2^ = 94%).

**Figure 4:**
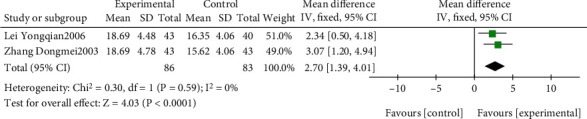
Forest plot of comparison: EGb versus drugs for VCI on MMSE levels.

The effect of EGb compared to DV on MMSE is outlined in Figure 4. We employed fixed effects models for pooling the effect estimates in two studies (*n* = 169 patients) [48, 57]. There was a significant difference in favor of EGb for improving MMSE (MD: 2.70; 95% CI: 1.39-4.01; *P* < 0.0001; *I*^2^ = 0%).

**Figure 5:**
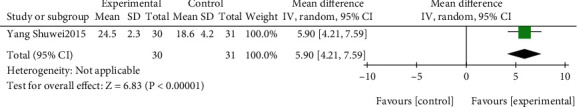
Forest plot of comparison: EGb combined with drugs for VCI versus blank group on MMSE levels.

The effects of EGb combined with DV compared to the blank on MMSE are summarized in Figure 5. Only one study (*n* = 61 patients) [56] investigated the effect of EGb in conjunction with DV. A significant difference was reported in favor of EGb in conjunction with DV to improve MMSE (MD: 5.90; 95% CI: 4.21-7.59; *P* < 0.00001).

**Figure 6:**
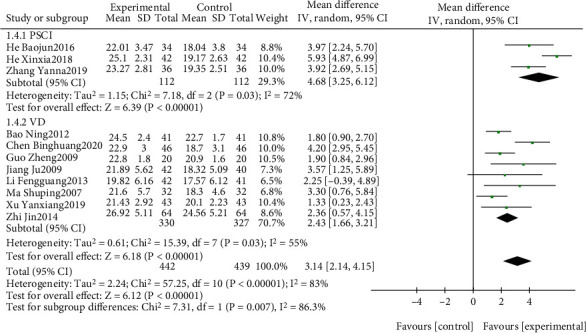
Forest plot of comparison: EGb combined with drugs for VCI versus drugs for VCI on MMSE levels (different types of cognitive impairment).

**Figure 7:**
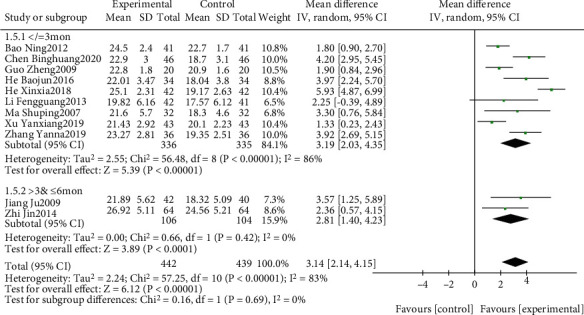
Forest plot of comparison: EGb combined with drugs for VCI versus drugs for VCI on MMSE levels (different treatment courses).

The effect of EGb combined with DV compared to DV alone on MMSE is summarized in Figures 6 and 7. We used random effects models for pooling the effect estimates from 11 trials (*n* = 881 patients) [38, 40, 43, 44, 46, 49–52, 54, 60]. There was a significant difference in favor of EGb with DV for improving MMSE (MD: 3.14; 95% CI: 2.14-4.15; *P* < 0.00001). However, the heterogeneity was substantial (*I*^2^ = 83%). Subgroup analysis was conducted according to the different types of cognitive impairment and different courses of intervention. There was a significant difference in favor of EGb with DV for improving MMSE in three studies concerning PSCI (MD: 4.68; 95% CI: 3.25-6.12; *P* < 0.00001; *I*^2^ = 72%) and in eight studies concerning VD (MD: 2.43; 95% CI: 1.66-3.21; *P* < 0.00001; *I*^2^ = 55%). There was a significant difference in favor of EGb with DV for improving MMSE in nine studies with three-month treatment courses (MD: 3.19; 95% CI: 2.03-4.35; *P* < 0.00001; *I*^2^ = 86%) and in two studies with six months of treatment (MD: 2.81; 95% CI: 1.40-4.23; *P* < 0.0001; *I*^2^ = 0%).

#### 3.4.2. Analysis of MoCA

**Figure 8:**
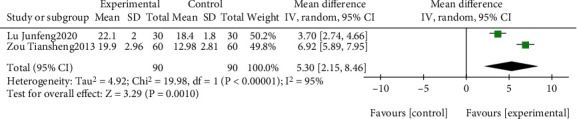
Forest plot of comparison: EGb versus blank group on MoCA levels.

The effect of EGb compared to the blank on MoCA is summarized in Figure 8. We used random effects models for pooling the effect estimates from two trials (*n* = 180 patients) [39, 59]. There was a significant difference in favor of EGb for improving MoCA (MD: 5.30; 95% CI: 2.15-8.46; *P* = 0.001). However, the heterogeneity was substantial (*I*^2^ = 95%).

**Figure 9:**
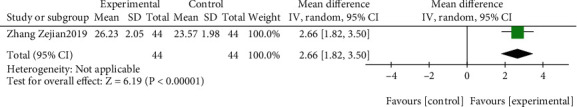
Forest plot of comparison: EGb combined with drugs for VCI versus blank group on MoCA levels.

The effect of EGb combined with DV compared to the blank on MoCA is summarized in Figure 9. Only one study (*n* = 88 patients) [58] investigated the effect of EGb in conjunction with DV. A significant difference was reported in favor of EGb in conjunction with DV to improve MoCA (MD: 2.66; 95% CI; 1.82-3.50; *P* < 0.00001).

**Figure 10:**
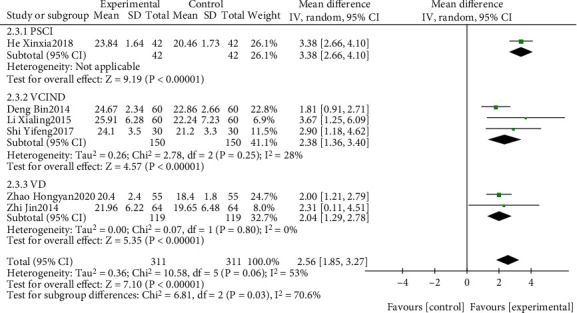
Forest plot of comparison: EGb combined with drugs for VCI versus drugs for VCI on MoCA levels (different types of cognitive impairment).

**Figure 11:**
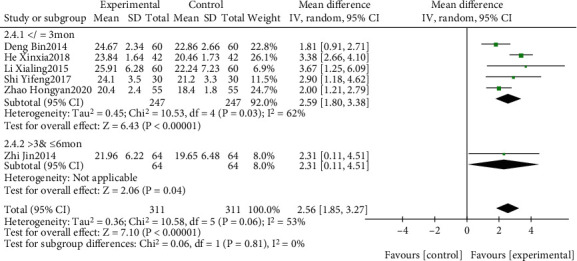
Forest plot of comparison: EGb combined with drugs for VCI versus drug treatment only for VCI on MoCA levels (different courses of treatment).

The effect of EGb combined with DV compared to DV alone on MoCA is summarized in Figures 10 and 11. We used random effects models for pooling the effect estimates from six trials (*n* = 622 patients) [42, 45–47, 50, 53]. There was a significant difference in favor of EGb with DV for improving MoCA levels (MD: 2.56; 95% CI: 1.85-3.27; *P* = 0.006; *I*^2^ = 53%). Subgroup analysis was conducted according to the different types of cognitive impairment and different courses of intervention. There was a significant difference in favor of EGb with DV for improving MoCA in one study concerning PSCI (MD: 3.38; 95% CI: 2.66-4.10; *P* < 0.00001) and in two studies concerning VD (MD: 2.04; 95% CI: 1.29-2.78; *P* < 0.00001; *I*^2^ = 0%). There was a significant difference in favor of EGb with DV for improving MoCA in five studies with three-month treatment courses (MD: 2.59; 95% CI: 1.80-3.38; *P* < 0.00001; *I*^2^ = 62%) and in one study with six months of treatment (MD: 2.31; 95% CI: 0.11-4.51; *P* = 0.04).

#### 3.4.3. Analysis of ADL

**Figure 12:**
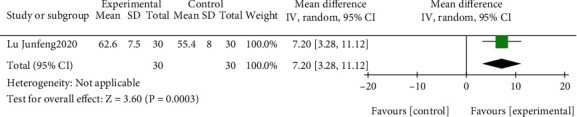
Forest plot of comparison: EGb versus blank group on ADL levels.

The effect of EGb compared to the blank on ADL is summarized in Figure 12. Only one trial (*n* = 60 patients) [39] reported a significant difference in favor of EGb for improving ADL (MD: 7.20; 95% CI: 3.28-11.12; *P* = 0.0003).

**Figure 13:**
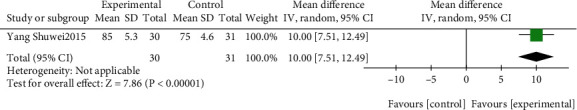
Forest plot of comparison: EGb combined with drugs for VCI versus blank group on ADL levels.

The effect of EGb combined with DV compared to the blank on ADL is summarized in Figure 13. Only one study (*n* = 61 patients) [56] investigated the effect of EGb in conjunction with DV. A significant difference was reported in favor of EGb in conjunction with DV to improve ADL (MD: 7.20; 95% CI: 3.28-11.12; *P* = 0.0003).

**Figure 14:**
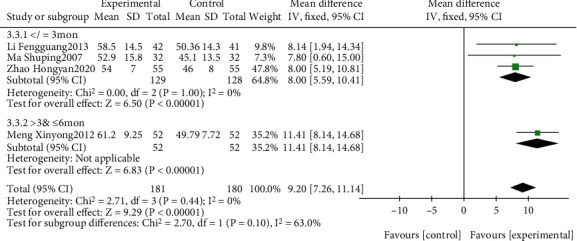
Forest plot of comparison: EGb combined with drugs for VCI versus drug treatment only for VCI on ADL levels (different treatments).

The effect of EGb combined with DV compared to DV on ADL is summarized in Figure 14. We used fixed effects models for pooling the effect estimates from four trials (*n* = 361 patients) [38, 40, 41, 45]. There was no significant difference between the two groups (MD: 9.20; 95% CI: 7.26-11.14; *P* = 0.44; *I*^2^ = 0%). Subgroup analysis was conducted according to different courses of intervention. There was a significant difference in favor of EGb with DV for improving ADL in five studies with three-month treatment courses (MD: 8.00, 95% CI: 5.59-10.41; *P* < 0.00001; *I*^2^ = 0%) and in one study with six months of treatment (MD: 11.41; 95% CI: 8.14-14.68; *P* < 0.00001).

#### 3.4.4. Analysis of HDS

**Figure 15:**
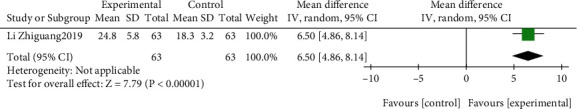
Forest plot of comparison: EGb versus blank group on HDS levels.

The effect of EGb compared to the blank on HDS is summarized in Figure 15. Only one trial (*n* = 126 patients) [55] reported that a significant difference in favor of EGb for improving HDS (MD: 6.50; 95% CI: 4.86-8.14; *P* < 0.00001).

**Figure 16:**
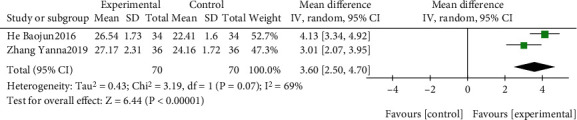
Forest plot of comparison: EGb combined with drugs for VCI versus drug treatment only for VCI on HDS levels.

The effect of EGb combined with DV compared to DV on HDS is summarized in Figure 16. We used random effects models for pooling the effect estimates from two trials (*n* = 140 patients) [44, 51]. There was a significant difference between the two groups (MD: 3.60; 95% CI: 2.50-4.70; *P* < 0.00001; *I*^2^ = 69%).

#### 3.4.5. Analysis of BI

**Figure 17:**
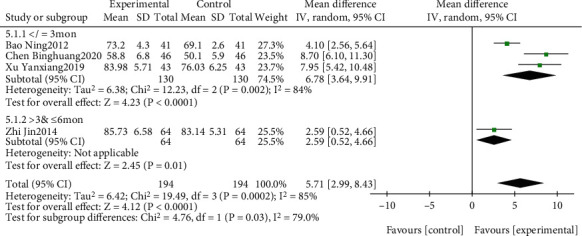
Forest plot of comparison: EGb combined with drugs for VCI versus drug treatment alone for VCI on BI levels (different treatments).

The effect of EGb combined with DV compared to DV alone on BI is summarized in Figure 17. We used random effects models for pooling the effect estimates from four trials (*n* = 388 patients) [43, 46, 54, 60]. There was a significant difference between the two groups (MD: 5.71; 95% CI: 2.99-8.43; *P* = 0.0002; *I*^2^ = 85%). Subgroup analysis was conducted according to different intervention courses. There was a significant difference in favor of EGb with DV for improving BI in three studies with three-month treatment courses (MD: 6.78; 95% CI: 3.64-9.91; *P* < 0.0001; *I*^2^ = 84%) and in one study with six months of treatment (MD: 2.59; 95% CI: 0.52-4.66; *P* = 0.01).

#### 3.4.6. Analysis of FAQ

**Figure 18:**
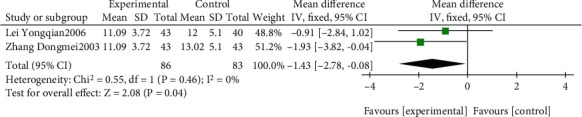
Forest plot of comparison: EGb versus drug treatment only for VCI on FAQ levels.

The effect of EGb compared to DV alone on FAQ is summarized in Figure 18. We used fixed effects models for pooling the effect estimates from two trials (*n* = 169 patients) [48, 57]. There was a significant difference in favor of EGb for improving FAQ (MD: -1.43; 95% CI: -2.78 to 0.08; *P* = 0.04; *I*^2^ = 0%).

**Figure 19:**
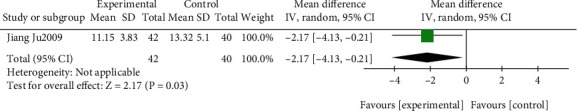
Forest plot of comparison: EGb combined with drugs for VCI versus drugs for VCI on FAQ levels.

The effect of EGb combined with DV compared to DV alone on FAQ is summarized in Figure 19. Only one study (*n* = 82 patients) [49] investigated the effect of EGb in conjunction with DV. A significant difference was reported in favor of EGb in conjunction with DV to improve FAQ (MD: -2.17; 95% CI: -4.13 to 0.21; *P* = 0.03).

### 3.5. Adverse Events

Adverse effects were reported in 15 studies [38–40, 43–45, 48–52, 54, 56, 57, 60] of the 23 included studies, but were not mentioned in the remaining eight studies [41, 42, 46, 47, 53, 55, 58, 59]. Five out of the 15 studies reported that no adverse effects occurred during the trial [38, 40, 45, 56, 60]. The remaining 10 studies [39, 41–44, 46–55, 57–59] reported nine different kinds of adverse events, in which methods for judging adverse events were participant and carer reports, medical notes, or clinical observation or a combination of these. These adverse events were reported in the treatment group and in the control group respectively, except one study which did not mention which group. Six studies reported both groups experienced dizziness/nausea/vomiting/insomnia and diarrhea [43, 44, 50, 51, 54, 57]. Two studies reoported skin rashes [54, 57] and one study reported dry mouth [49] happened in the treatment group, while one study reported gingival bleeding [39] and one study [54] reported elevated transaminase happened in the control group. However, life-threatening adverse effects were not reported in any of the studies. All the details of the reported adverse effects are described in the last column in Table 1.

### 3.6. Publication Bias

**Figure 20:**
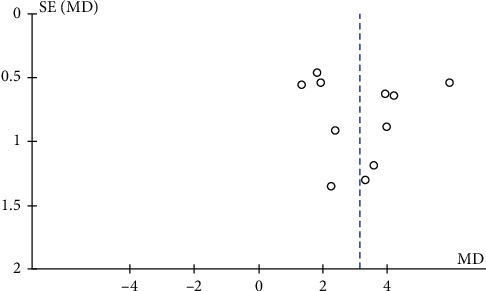
Funnel plot of comparison of EGb with drugs for VCI versus drugs alone on MMSE. The horizontal axis shows the mean difference between the estimated effects of EGb with drugs versus drugs alone on MMSE, while the vertical axis shows the standard error of the intervention effect on a reversed scale.

To explore the issue of publication bias, a funnel plot was constructed in which the standard error of the mean difference was plotted against the mean difference. The funnel plots for the MMSE from 11 studies [38, 40, 43, 44, 46, 49–52, 54, 60] that compared EGb with DV versus DV alone suggested publication bias as many of the smaller studies had more positive results (Figure 20).

### 3.7. Grade Evaluation of Outcomes

GRADEpro software, version 3.6.1, was used to evaluate the quality of evidence of the outcomes. Due to various biases, inconsistencies, inaccuracies, and publication bias, the evidence of certainty ranges from medium certainty to very low certainty, as shown in Table 2.

## 4. Discussion

There is currently no early intervention strategy for cognitive impairment and preventing cognitive decline [61]. The interests of the public and the medical profession in the use of EGb for cognitive impairment and dementia have grown considerably in recent years [8, 61, 62]. There is evidence to support the efficacy of EGb treatment for dementia, and the effect has been found to be dose- and age-dependent [32]. Our analysis supports the efficacy of EGb (in tablet form) for VCI, and it appeared to be well tolerated. This study is a systematic review of the English and Chinese literature to determine the efficacy and safety of EGb for VCI. Twenty-three RCTs including a total of 2019 patients with VCI met the inclusion criteria. The main finding of this review was that EGb treatment appears to be more effective than controls as assessed by various measures of cognitive function, including MMSE, MoCA, HDS, ADL, BI, and FAQ. The evidence of certainty ranges from medium certainty to very low certainty. Although the findings appear positive, the poor methodological quality and clinical heterogeneity of the included studies limit the evidence to support the use of EGb for VCI. In addition, the safety of EGb treatment could not be confirmed because only 65% (15/23) of the studies mentioned safety issues or investigated adverse effects. Due to the limited number of included studies that analyzed safety, we failed to draw a definitive conclusion, which is one of the major issues needing further confirmation. More attention should be paid to both the monitoring and reporting of adverse effects of EGb.

There are some limitations in this study: (1) Only Chinese and English literature was included. All the participants were Chinese, and the administration of EGb was limited to tablets. This could affect the generalization of the results. (2) The data analysis was performed using published trials with positive results, suggesting that trials with negative results may have been missed, which would make the true effect substantially different from the estimate of the effect. (3) The quality of the included trials was generally low, and the certainty of evidence ranged from medium certainty to very low certainty, reducing our confidence in the estimates of the effects. Neither intention analysis nor allocation concealment strategy was mentioned in any of the studies. (4) We put the different kinds of VCI together for each outcome evaluation, although we performed subgroup analysis on the different VCI type as a supplementary analysis. This may not be in line with clinical practice. (5) The treatment courses in most of the included studies were relatively short, and the long-term consequences of EGb treatment for VCI remain unexplored. (6) Clinicians differ in their experience and use of the measurement scales, such as MMSE, MoCA, and ADL. Inconsistent treatment methods in the control group and differences in drug dosage or course may also have an impact on the evaluation of efficacy and safety. The in-homogeneity of the basic supporting treatment may have a confounding effect. Consequently, the results generated from the current review should be interpreted with caution.

Conducting clinical trials in VCI has many obstacles. Clinical outcomes in VCI patients are multifaceted, as they may experience further cognitive decline and may also experience progressive vascular morbidity, mortality, and general deterioration of function [15, 63, 64]. Outcome measures in future trials should include brain structure and function imaging and disease progression determined by macro network diagrams of the brain along with patient-reported outcomes, such as PET (positron emission tomography) and SPECT (single-photon emission computed tomography), measured using a validated rCBF (regional cerebral blood flow) scale [65–67]. In addition, the 23 included studies contained various types of VCI and different kinds of DV. VCI encompasses a heterogeneous population in terms of cognitive profile and severity of deficits, vascular brain injury, and concurrent neurodegenerative pathology [9]. Thus, the patients should be divided into specific subgroups according to different ages and EGb dosages. It is reasonable to take the most common kinds of VCI with high incidence as future target types to explore the precise benefits obtained from EGb. A long follow-up with long-term outcomes is important to determine the effectiveness and safety of EGb[68]. The safety of EGb is a major concern in clinical practice. Thus, safety monitoring of EGb in pharmacovigilance systems is needed.

Our results are based on published studies, the number of included studies was small, and the quality was poor, which may lead to low credibility of the conclusions. Future research on EGb in VCI should implement higher quality research methodology to limit the potential for bias. More large-scale, multicenter randomized controlled clinical trials on related mechanisms should be implemented in a scientifically designed manner, clinically important outcomes should be selected, and longer treatments and follow-up periods should be used. We recommend that the SPIRIT 2013 and the CONSORT 2010 statement [69–72] should be used as a guideline when designing and reporting RCTs for EGb in the future.

## 5. Conclusion

In summary, in patients with VCI, *Ginkgo biloba* extract tablets can be taken separately or in addition to other medication. Although the available evidence from the present review supported the efficacy of *Gingko biloba* extract, recommendations for its routine use for the treatment of VCI are limited by the poor methodological quality and clinical heterogeneity of the included studies. Nevertheless, we have identified an area that is worthy of further study. Further well-designed rigorous RCTs of *Ginkgo biloba* extract for VCI are needed.

## Figures and Tables

**Table 1:** Characteristics of the included studies.

Study ID	Types of cognitive impairment	Sample size		Interventions		Treatment course/month	Outcomes	Adverse events
		T	C	T	C			
[50]	PSCI	42	42	EGb 9.6 mg tid + donepezil 5 mg qn	Donepezil 5 mg qn	3	①②	The treatment group had 3 cases with mild dizziness. In the control group, 1 patient had mild dizziness and 1 patient had poor appetite
[51]	PSCI	34	34	EGb 19.2 mg tid + donepezil 5 mg qn	Donepezil 5 mg qn	3	①④	One patient in the treatment group had dizziness/nausea/poor appetite. The control group had 7 patients with dizziness/nausea/poor appetite
[44]	PSCI	36	36	EGb 19.2 mg tid + donepezil 5 mg qn	Donepezil 5 mg qn	3	①④	In the treatment group, 1 case had anorexia, 1 case had dizziness, 2 cases had insomnia, and 2 cases had diarrhea. In the control group, there were 2 cases with loss of appetite, 1 case with nausea and vomiting, 2 cases with dizziness, 1 case with insomnia, and 1 case with diarrhea
[38]	VD	42	41	EGb 40 mg tid + nimodipine 30 mg tid	Nimodipine 30 mg tid	3	①③	-
[40]	VD	32	32	EGb 0.5 g tid + huperzine 100 mg bid	Piracetam 1.2 g tid	2	①③	-
[52]	VD	20	20	EGb 19.2 mg tid + donepezil 5 mg qn	Donepezil 5 mg qn	3	①	3 cases with nausea/loss of appetite and 2 cases with dizziness
[60]	VD	41	41	EGb 19.2 mg tid + donepezil 5 mg qn	Donepezil 5 mg qn	3	①⑤	-
[39]	VD	30	30	EGb 24 mg tid	Conventional treatment	3	①②③	Nausea was observed in 2 patients in the treatment group In the control group, 2 patients had nausea and 1 patient had gingival bleeding
[58]	VD	44	44	EGb 19.2 mg tid + oxiracetam 800 mg bid	Conventional treatment	3	②	Not reported
[55]	VD	63	63	EGb 19.2 mg tid	Conventional treatment	2	①④	Not reported
[41]	VD	52	52	EGb 1piece tid + piracetam 3 piece tid	Piracetam 1.2 g tid	6	③	Not reported
[54]	VD	46	46	EGb 19.2 mg tid + butylphthalide 0.2 g bid	Butylphthalide 0.2 g bid	2	①⑤	In the treatment group, there were 2 cases with dizziness, 1 case with nausea and vomiting, and 1 case with rash. The control group had 1 case with dizziness, 1 case with nausea and vomiting, and 1 case with elevated transaminase
[45]	VD	55	55	EGb 19.2 mg tid + donepezil 5 mg qn	Donepezil 5 mg qn	3	②③	-
[43]	VD	43	43	EGb 19.2 mg tid + donepezil 5 mg qn	Donepezil 5 mg qn	3	①⑤	In the treatment group, there were 1 case with dizziness, 3 cases with nausea, and 1 case with abdominal distension. The control group had 1 case with dizziness and 1 case with nausea
[42]	VCI	30	30	EGb 40 mg tid + donepezil 5 mg qn	Donepezil 5 mg qn	6	②	Not reported
[47]	VCIND	60	60	EGb 19.2 mg tid + nimodipine 30 mg tid	Nimodipine 30 mg tid	3	②	Not reported
[53]	VCIND	60	60	EGb 19.2 mg tid + nimodipine 30 mg tid	Nimodipine 30 mg tid	2	②	Not reported
[49]	VD	42	40	EGb 2 pieces tid + ergoloid mesylate sustained release capsules, 1 grain bid	Piracetam 0.8 g tid	6	①⑥	In the treatment group, there were 2 cases with nausea, 3 cases with dry mouth, and 1 case with dizziness
[46]	VD	64	64	EGb 2 pieces tid + huperzine 0.1 mg tid	Huperzine 0.1 mg tid	6	①②⑤	Not reported
[56]	PSCI	30	31	EGb 19.2 mg tid + XueSaiTong JiaoNang 0.2 g tid	Conventional treatment	2	①③	-
[48]	VD	43	40	EGb 0.4 g tid	Flunarizine hydrochloride 5 mg qd	6	①⑥	The treatment group had 2 cases with dizziness. The control group had 17 patients with headache/dizziness/drowsiness
[57]	VD	43	43	EGb 80 mg tid	Piracetam 0.8 g tid	6	①⑥	In the treatment group, there were 2 cases with rash and 2 cases with dizziness
[59]	VCIND	60	60	EGb 19.2 mg tid	Conventional treatment	3	②	Not reported

T: treatment group; C: control group; PSCI: cognitive impairment after cerebral infarction; VD: vascular dementia; VCIND: vascular cognitive impairment without dementia; ①: MMSE; ②: MoCA; ③: ADL; ④: HDS; ⑤: BI; ⑥: FAQ; -: no adverse reactions.

**Table 2:** Summary of findings.

EGb alone or combined with drugs for VCI versus drugs for VCI alone or blank group “Drugs for VCI” and “blank” are abbreviated as “DV” and “B,” respectively.					

Patients or population: the patients diagnosed with VCI were not limited in terms of age, sex, and race Intervention: use of EGb alone or combined with drugs for VCI Comparison: drugs for VCI or blank control					

Outcomes	Anticipated absolute effects^∗^ (95% CI)		Relative effect (95% CI)	Number of participants (studies)	Quality of the evidence (GRADE)
	Risk with control group	Risk with experimental group			
MMSE					
EGb vs. B	MMSE mean range 20.1-21.5	MD 3.04 (0.10 lower to 5.98 higher)	—	186 (2 RCTs)	⊕⊝⊝⊝ Very low^1,2,3,4^
EGb vs. DV	The average levels in the control group were 15.62-16.35	MD 2.7 (1.39 lower to 4.01 higher)	—	169 (2 RCTs)	⊕⊝⊝⊝ Very low^1,2,4^
EGb + DV vs. B	There was a significant difference in MMSE between EGb + DV and B (*P* < 0.00001) in one RCT [56].		—	61 (1 RCT)	⊕⊝⊝⊝ Low^1,2^
EGb + DV vs. DV	MMSE range 17.57-24.56	MD 3.14 (0 lower to 4.15 higher)	—	881 (11 RCTs)	⊕⊝⊝⊝ Very low^1,3,5^
MoCA					
EGb vs. B	MoCA mean range 12.98-18.4	MD 5.3 (2.15 lower to 8.46 higher)	—	180 (2 RCTs)	⊕⊝⊝⊝ Very low^1,2,3,4^
EGb + DV vs. B	There was a significant difference in MMSE between EGb + DV and B (*P* < 0.00001) in one RCT [58]		—	88 (1 RCT)	⊕⊝⊝⊝ Low^1,2^
EGb + DV vs. DV	MoCA mean range 18.4-22.86	MD 2.56 (2.66 lower to 3.27 higher)	—	622 (6 RCTs)	⊕⊝⊝⊝ Very low^1,3,5^
ADL					
EGb vs. B	There was a significant difference in ADL between EGb and B (*P* = 0.0003) in one RCT [39]		—	60 (1 RCT)	⊕⊝⊝⊝ Very low^1,2,4^
EGb + DV vs. B	There was a significant difference in ADL between EGb + DV and B (*P* < 0.00001) in one RCT [56]		—	61 (1 RCTs)	⊕⊝⊝⊝ Very low^1,2,4^
EGb + DV vs. DV	ADL mean range 45.1-50.36	MD 9.2 (5.59 lower to 11.41 higher)	—	361 (4 RCTs)	⊕⊝⊝⊝ Medium^1^
HDS					
EGb vs. B	There was a significant difference in HDS between EGb and B (*P* < 0.00001) in one RCT [55]		—	126 (1 RCT)	⊕⊝⊝⊝ Very low^1,2,4^
EGb + DV vs. DV	HDSL mean range 22.41-24.16	MD 3.6 (2.5 lower to 4.7 higher)	—	140 (2 RCTs)	⊕⊝⊝⊝ Very low^1,2,3^
BI					
EGb + DV vs. DV	BI mean range 50.1-83.14	MD 5.71 (3.64 lower to 8.43 higher)	—	388 (4 RCTs)	⊕⊝⊝⊝ Very low^1,3,4,5^
FAQ					
EGb vs. DV	FAQ mean range 12-13.02	MD1.43 (2.78 higher to 0.08 lower)	—	169 (2 RCTs)	⊕⊝⊝⊝ Very low^1,2^
EGb + DV vs. DV	There was a significant difference in FAQ between EGb + DV and DV (*P* = 0.03) in one RCT [49]		—	82 (1 RCT)	⊕⊝⊝⊝ Very low^1,2^

^∗^The risk in the intervention group (and its 95% confidence interval) is based on the assumed risk in the comparison group and the relative effect of the intervention (and its 95% CI). MMSE: Mini-Mental State Examination; MoCA: Montreal Cognitive Assessment; CI: confidence interval; RCT: randomized controlled trials. ^1^Bias in the integrity of hidden allocation, blind method, and result data. ^2^Funnel plot asymmetry. ^3^High heterogeneity, *I*^2^ ≥ 50%. ^4^Wide confidence interval. ^5^Differences in the types of cognitive impairment of the subjects, control medication, or course of treatment. Outcomes: EGb vs. B: EGb vs. blank control; EGb vs. DV: EGb vs. drug for VCI; EGb + DV vs. B: EGb + drug for VCI vs. blank control; EGb + DV vs. DV: EGb + drug for VCI vs. drug for VCI.

## Data Availability

All relevant data are within the article and its supporting information files. The data supporting this systematic review and meta-analysis are from published literature and accessible datasets, which have been cited. The original included articles used in this study are available from the corresponding author upon request.

## Supplementary Materials

Supplementary 1 Appendix 1: checklist of items for the meta-analysis according to PRISMA statement.

Supplementary 2 Appendix 2: supplementary retrieval information: RCTs of Ginkgo biloba Extract in the treatment of VCI.
