# Supplementary material for: EGb in the Treatment for Patients with VCI: A Systematic Review and Meta-Analysis
Source: Oxid Med Cell Longev. 2021 Aug 27;2021:8787684. doi: 10.1155/2021/8787684 (PMC8422158; doi:10.1155/2021/8787684)
Supplement: Supplementary 2 — Appendix 2: supplementary retrieval information: RCTs of Ginkgo biloba Extract in the treatment of VCI. [file 8787684.f2.pdf]

1. Literature search

1.1 Summary of literature retrieval

A total of 7 databases and 2 trial registries were searched. Four Chinese databases (CNKI, Wanfang, VIP, and Sinomed), three English databases (PubMed, Cochrane Library, and Embase), and two major trial registries (ChiCTR and ClinicalTrials.gov), retrieved a total of 61007 entries. Actually import NE software 57821.See Table 1 for details.

Table 1. Literature retrieval sources and quantity summary table of traditional Chinese medicine in the treatment of vascular cognitive impairment

Source	Database	Amount of literature	Actual imported NE quantity (after reduplication)
Chinese databases	CNKI	5999	5810
	Wanfang	4464	4413
	VIP	5200	4790
	Sinomed	38834	36346
English databases	Pubmed	2438	2432
	Cochrane library	855	836
	Embase	3216	3191
Clinical Trial Registry	ChiCTR	0	0
	ClinicalTrials.gov	1	0
**A total of**		**61007**	**57821**

2. Each database retrieval strategy

2.1 Selection of search terms

2.1.1 Four Chinese databases: CNKI/Wanfang/VIP/Sinomed(CBM)

（1）Disease related:

Vascular cognitive impairment, mild vascular cognitive impairment, mild, severe vascular cognitive dysfunction of vascular cognitive impairment and severe vascular

cognitive dysfunction, vascular dementia, poststroke dementia, subcortical ischemic vascular dementia, multiple infarction dementia, mixed dementia risk factors, the correlation between vascular cognitive impairment, ischemic vascular cognitive impairment, hemorrhagic blood, tube of cognitive impairment, other cerebrovascular disease vascular cognitive impairment, cerebrovascular disease with AD, not sexual vascular cognitive impairment, dementia, vascular dementia cognitive dysfunction, cognitive impairment after stroke, cognitive impairment after stroke, cerebral vascular cognitive impairment, lacunar infarction cognitive dysfunction, cognitive impairment after stroke, brain white matter loose cognitive impairment after stroke, dementia,

Ischemic cognitive impairment, multiple infarct dementia, small vascular dementia, strategic site infarct dementia, hypoperfusion dementia, hemorrhagic dementia, autosomal dominant cerebral arteriopathy with subcortical infarction and leukoencephalopathy, subcortical vascular cognitive impairment (SVCI), artery atherosclerosis sex cognitive impairment of cognitive impairment, moyamoya disease, cognitive impairment, cognitive impairment, cognitive dysfunction, cognitie impairment, cognitive impairment, mild cognitive impairment, cognitive decline, diabetic cognitive dysfunction, forgotten mild cognitive impairment, the memory type of mild cognitive impairment, mild cognitive type Cognitive impairment, non-dementia cognitive impairment, idiotic disease, idiotic syndrome, amnesia, amnesia, idiocy, inwisdom, wai, dementia, stupid, stupid syndrome, brain marrow disappear, language disorder

（2）Chinese medicine treatment measures related:

Chinese and western medicine, Chinese medicine, Chinese medicine, proprietary Chinese medicine, Chinese herbal medicine, herbal medicine, and the formulas compound, acupuncture, acupuncture, needle head, earpins, body acupuncture, cupping, plum needle, needle, bleeding, blood, collaterals, acupuncture point, scrapping, massage, cupping and moxibustion, massage, qi gong, tai chi, eight brocade, stick, medicine bath, foot bath, acupoint injection, combined with medicine and complementary alternative medicine, alternative medicine and traditional

medicine, cience, ethnic medicine, soup, tablet, pill, powder, capsule, granule, liquid, mixture, water, paste, granule, injection, Dan, wine, tea, dew

2.1.2 Four major English databases

（1）Pubmed

①Disease-related Mesh words:

1）Cognitive dysfunction

Synonym：Cognitive Dysfunctions; Dysfunction, Cognitive; Dysfunctions, Cognitive; Cognitive Impairments; Cognitive Impairment；Impairment, Cognitive; Impairments, Cognitive; Mild Cognitive Impairment; Cognitive Impairment, Mild; Cognitive Impairments, Mild; Impairment, Mild Cognitive; Impairments, Mild Cognitive; Mild Cognitive Impairments; Mild Neurocognitive Disorder; Disorder, Mild Neurocognitive; Disorders, Mild Neurocognitive; Mild Neurocognitive Disorders;Neurocognitive Disorder, Mild; Neurocognitive Disorders, Mild; Cognitive Decline; Cognitive Declines; Decline, Cognitive; Declines, Cognitive; Mental Deterioration; Deterioration, Mental; Deteriorations, Mental; Mental Deteriorations

2）Dementia, vascular

Synonym： Dementias, Vascular; Vascular Dementias; Vascular Dementia; Vascular Dementia, Acute Onset; Acute Onset Vascular Dementia; Subcortical Vascular Dementia; Dementia, Subcortical Vascular; Dementias, Subcortical Vascular; Subcortical Vascular Dementias; Vascular Dementia, Subcortical; Vascular Dementias, Subcortical; Arteriosclerotic Dementia; Arteriosclerotic Dementias; Dementia, Arteriosclerotic; Dementias, Arteriosclerotic; Binswanger Disease; Disease, Binswanger; Chronic Progressive Subcortical Encephalopathy; Binswanger Encephalopathy; Leukoencephalopathy, Subcortical; Leukoencephalopathies, Subcortical; Subcortical Leukoencephalopathies; Encephalopathy, Subcortical Arteriosclerotic; Binswanger's Disease; Binswangers Disease; Disease, Binswanger's; Encephalopathy, Subcortical, Chronic Progressive; Subcortical Encephalopathy, Chronic Progressive; Subcortical Leukoencephalopathy; Subcortical Arteriosclerotic Encephalopathy; Arteriosclerotic Encephalopathy, Subcortical; Arteriosclerotic Encephalopathies, Subcortical; Encephalopathies, Subcortical Arteriosclerotic;

Subcortical Arteriosclerotic Encephalopathies; Encephalopathy, Binswanger's; Binswanger's Encephalopathy; Encephalopathy, Binswangers; Encephalopathy, Binswanger; Encephalopathy, Chronic Progressive Subcortical

3）Dementia, Multi-Infarct

Synonym: Dementia, Multi Infarct; Dementias, Multi-Infarct;Multi-Infarct Dementias; Dementia Multi-Infarct; Dementia Multi Infarct; Dementia Multi-Infarcts; Multi-Infarct, Dementia; Multi-Infarcts, Dementia; Dementia, Multiinfarct; Dementias, Multiinfarct; Multiinfarct Dementia; Multiinfarct Dementias; Multi-Infarct Dementia; Multi Infarct Dementia; Lacunar Dementia; Dementia, Lacunar; Dementias, Lacunar; Lacunar Dementias

4）Words other than the above:

Vascular cognitive impairment; Vascular cognitive disorders; Vascular neurocognitive disorders; Vascular cognitive disorders; Vascular neurocognitive disorder; Vascular mild cognitive impairment; Vascular cognitive impairment no dementia; Post stroke cognitive impairment; Post stroke dementia; Subcortical vascular dementia; Cognitive impairment no dementia because of cerebrovascular disease; Ischemic vascular dementia; Subcortical vascular disease; Primary degenerative dementia; Mixed dementia; Cerebrovascular cognitive impairment; Subcortical ischemic vascular disease

②Chinese medicine treatment related:

1）Mesh of TCM：Medicine, Chinese Traditional

Synonyms： Traditional Chinese Medicine; Chung I Hsueh; Hsueh, Chung I; Traditional Medicine, Chinese; Zhong Yi Xue; Chinese Traditional Medicine; Chinese Medicine, Traditional; Traditional Tongue Diagnosis; Tongue Diagnoses, Traditional; Tongue Diagnosis, Traditional; Traditional Tongue Diagnoses; Traditional Tongue Assessment; Tongue Assessment, Traditional; Traditional Tongue Assessments; medicine, Chinese traditional

2）Mesh of Chinese herbal medicine：Herbal Medicine

Synonyms： Medicine, Herbal; Hawaiian Herbal Medicine; Hawaiian Herbal Medicines; Herbal Medicine, Hawaiian ; Herbal Medicines, Hawaiian ; Medicine,

Hawaiian Herbal; Medicines, Hawaiian Herbal ; La'au Lapa'au ; Laau Lapaau ; La au Lapa au; Herbalism

3）Mesh of Traditional medicine：Medicine, Traditional

Synonyms： Traditional Medicine; Home Remedies; Home Remedy; Remedies, Homeziy; Remedy, Home; Medicine, Primitive; Primitive Medicine; Medicine, Folk; Folk Medicine; Medicine, Indigenous; Indigenous Medicine; Folk Remedies; Folk Remedy; Remedies, Folk; Remedy, Folk; Ethnomedicine

4）Mesh of replacement therapy：Complementary Therapies

Synonyms： Therapies, Complementary; Therapy, Complementary; Complementary Medicine; Medicine, Complementary; Alternative Medicine; Medicine, Alternative; Alternative Therapies; Therapies, Alternative; Therapy, Alternative

5）Mesh of acupuncture：Acupuncture

Synonyms： Pharmacopuncture; Acupuncture Treatment; Acupuncture Treatments; Treatment, Acupuncture; Therapy, Acupuncture; Pharmacoacupuncture Treatment; Treatment, Pharmacoacupuncture; Pharmacoacupuncture Therapy; Therapy, Pharmacoacupuncture; Acupotomy; Acupotomies; Acupunctures, Ear; Ear Acupunctures; Auricular Acupuncture; Ear Acupuncture; Acupuncture, Auricular; Acupunctures, Auricular; Auricular Acupunctures; Acupuncture Point; Point, Acupuncture; Points, Acupuncture; Acupoints; Acupoint; Analgesia, Acupuncture; Acupuncture Anesthesia; Anesthesia, Acupuncture

6）Mesh of massage：Moxibustion；Massage

Synonyms：Moxabustion; Zone Therapy; Therapies, Zone; Zone Therapies; Therapy, Zone; Massage Therapy; Massage Therapies; Therapies, Massage; Therapy, Massage

7）Mesh of cupping therapy：Cupping Therapy

Synonyms： Cupping Therapies; Therapy, Cupping; Cupping Treatment; Cupping Treatments; Treatment, Cupping; baguan

8）Mesh of Qigong：Qigong

Synonyms：Qi Gong; Ch'i Kung

9）Mesh of Tai Ji：Tai Ji

Synonyms： Tai-ji; Tai Chi; Chi, Tai; Tai Ji Quan; Ji Quan, Tai; Quan, Tai Ji; Taiji; Taijiquan; T'ai Chi; Tai Chi Chuan

10）TCM treatment in addition to the above words other words:

Baduanjin; Tuina

（2）Cochrane library

①Disease-related Mesh words:

Cognitive dysfunction

Synonyms ： Cognitive Dysfunctions; Dysfunction, Cognitive; Dysfunctions, Cognitive; Cognitive Impairments; Cognitive Impairment; Impairment, Cognitive; Impairments, Cognitive; Mild Cognitive Impairment; Cognitive Impairment, Mild; Cognitive Impairments, Mild; Impairment, Mild Cognitive; Impairments, Mild Cognitive; Mild Cognitive Impairments; Mild Neurocognitive Disorder; Disorder, Mild Neurocognitive; Disorders, Mild Neurocognitive; Mild Neurocognitive Disorders; Neurocognitive Disorder, Mild; Neurocognitive Disorders, Mild; Cognitive Decline; Cognitive Declines; Decline, Cognitive; Declines, Cognitive; Mental Deterioration; Deterioration, Mental; Deteriorations, Mental; Mental Deteriorations

2）Dementia, vascular

Synonyms： Dementias, Vascular; Vascular Dementias; Vascular Dementia; Vascular Dementia, Acute Onset; Acute Onset Vascular Dementia; Subcortical Vascular Dementia; Dementia, Subcortical Vascular; Dementias, Subcortical Vascular; Subcortical Vascular Dementias; Vascular Dementia, Subcortical; Vascular Dementias, Subcortical; Arteriosclerotic Dementia; Arteriosclerotic Dementias; Dementia, Arteriosclerotic; Dementias, Arteriosclerotic; Binswanger Disease; Disease, Binswanger; Chronic Progressive Subcortical Encephalopathy; Binswanger Encephalopathy; Leukoencephalopathy, Subcortical; Leukoencephalopathies, Subcortical; Subcortical Leukoencephalopathies; Encephalopathy, Subcortical Arteriosclerotic; Binswanger's Disease; Binswangers Disease; Disease, Binswanger's; Encephalopathy, Subcortical, Chronic Progressive; Subcortical Encephalopathy, Chronic Progressive; Subcortical Leukoencephalopathy; Subcortical Arteriosclerotic

Encephalopathy; Arteriosclerotic Encephalopathy, Subcortical; Arteriosclerotic Encephalopathies, Subcortical; Encephalopathies, Subcortical Arteriosclerotic; Subcortical Arteriosclerotic Encephalopathies; Encephalopathy, Binswanger's; Binswanger's Encephalopathy; Encephalopathy, Binswangers; Encephalopathy, Binswanger; Encephalopathy, Chronic Progressive Subcortical

3）Dementia, Multi-Infarct

Synonyms： Dementia, Multi Infarct; Dementias, Multi-Infarct; Multi-Infarct Dementias; Dementia Multi-Infarct; Dementia Multi Infarct; Dementia Multi-Infarcts; Multi-Infarct, Dementia; Multi-Infarcts, Dementia; Dementia, Multiinfarct; Dementias, Multiinfarct; Multiinfarct Dementia; Multiinfarct Dementias; Multi-Infarct Dementia; Multi Infarct Dementia; Lacunar Dementia; Dementia, Lacunar; Dementias, Lacunar; Lacunar Dementias

4）Words other than the above:

Vascular cognitive impairment; Vascular cognitive disorders; Vascular neurocognitive disorders; Vascular cognitive disorders; Vascular neurocognitive disorder; Vascular mild cognitive impairment; Vascular cognitive impairment no dementia; Post stroke cognitive impairment; Post stroke dementia; Subcortical vascular dementia; Cognitive impairment no dementia because of cerebrovascular disease; Ischemic vascular dementia; Subcortical vascular disease; Primary degenerative dementia; Mixed dementia; Cerebrovascular cognitive impairment; Subcortical ischemic vascular disease

②Chinese medicine treatment related:

1）Mesh of TCM：Medicine, Chinese Traditional

Synonyms： Traditional Chinese Medicine; Chung I Hsueh; Hsueh, Chung I; Traditional Medicine, Chinese; Zhong Yi Xue; Chinese Traditional Medicine; Chinese Medicine, Traditional; Traditional Tongue Diagnosis; Tongue Diagnoses, Traditional; Tongue Diagnosis, Traditional; Traditional Tongue Diagnoses; Traditional Tongue Assessment; Tongue Assessment, Traditional; Traditional Tongue Assessments; medicine, Chinese traditional

2）Mesh of Chinese herbal medicine：Herbal Medicine

Synonyms： Medicine, Herbal; Hawaiian Herbal Medicine ; Hawaiian Herbal Medicines; Herbal Medicine, Hawaiian ; Herbal Medicines, Hawaiian ; Medicine, Hawaiian Herbal; Medicines, Hawaiian Herbal ; La'au Lapa'au ; Laau Lapaau ; La au Lapa au; Herbalism

3）Mesh of Traditional medicine：Medicine, Traditional

Synonyms： Traditional Medicine; Home Remedies; Home Remedy; Remedies, Homeziy; Remedy, Home; Medicine, Primitive; Primitive Medicine; Medicine, Folk; Folk Medicine; Medicine, Indigenous; Indigenous Medicine; Folk Remedies; Folk Remedy; Remedies, Folk; Remedy, Folk; Ethnomedicine

4）Mesh of acupuncture：Acupuncture

Synonyms： Pharmacopuncture; Acupuncture Treatment; Acupuncture Treatments; Treatment, Acupuncture; Therapy, Acupuncture; Pharmacoacupuncture Treatment; Treatment, Pharmacoacupuncture; Pharmacoacupuncture Therapy; Therapy, Pharmacoacupuncture; Acupotomy; Acupotomies; Acupunctures, Ear; Ear Acupunctures; Auricular Acupuncture; Ear Acupuncture; Acupuncture, Auricular; Acupunctures, Auricular; Auricular Acupunctures; Acupuncture Point; Point, Acupuncture; Points, Acupuncture; Acupoints; Acupoint; Analgesia, Acupuncture; Acupuncture Anesthesia; Anesthesia, Acupuncture

5）Mesh of massage：Moxibustion；Massage

Synonyms：Moxabustion; Zone Therapy; Therapies, Zone; Zone Therapies; Therapy, Zone; Massage Therapy; Massage Therapies; Therapies, Massage; Therapy, Massage

6）Mesh of cupping therapy：Cupping Therapy

Synonyms： Cupping Therapies; Therapy, Cupping; Cupping Treatment; Cupping Treatments; Treatment, Cupping; baguan

7）Mesh of Qigong：Qigong

Synonyms：Qi Gong; Ch'i Kung

8）Mesh of Tai Ji：Tai Ji

Synonyms：Tai-ji; Tai Chi; Chi, Tai; Tai Ji Quan; Ji Quan, Tai; Quan, Tai Ji; Taiji; Taijiquan; T'ai Chi; Tai Chi Chuan

9）TCM treatment in addition to the above words other words:

Complementary Therapies; Therapies, Complementary; Therapy, Complementary; Complementary Medicine; Medicine, Complementary; Alternative Medicine; Medicine, Alternative; Alternative Therapies; Therapies, Alternative; Therapy, Alternative; Baduanjin; Tuina

（3）Embase:

①Disease-related

1）Disease related Mesh: Multi infarct dementia

Synonyms: Dementia, Multi Infarct; Dementias, Multi-Infarct; Multi-Infarct Dementias; Dementia Multi-Infarct; Dementia Multi Infarct; Dementia Multi-Infarcts; Multi-Infarct, Dementia; Multi-Infarcts, Dementia; Dementia, Multiinfarct; Dementias, Multiinfarct; Multiinfarct Dementia; Multiinfarct Dementias; Multi-Infarct Dementia; Multi Infarct Dementia; Lacunar Dementia; Dementia, Lacunar; Dementias, Lacunar; Lacunar Dementias

2）Words other than the above:

Vascular dementia; Dementias, Vascular; Vascular Dementias; Vascular Dementia; Vascular Dementia, Acute Onset; Acute Onset Vascular Dementia; Subcortical Vascular Dementia; Dementia, Subcortical Vascular; Dementias, Subcortical Vascular; Subcortical Vascular Dementias; Vascular Dementia, Subcortical; Vascular Dementias, Subcortical; Arteriosclerotic Dementia; Arteriosclerotic Dementias; Dementia, Arteriosclerotic; Dementias, Arteriosclerotic; Binswanger Disease; Disease, Binswanger; Chronic Progressive Subcortical Encephalopathy; Binswanger Encephalopathy; Leukoencephalopathy, Subcortical; Leukoencephalopathies, Subcortical; Subcortical Leukoencephalopathies; Encephalopathy, Subcortical Arteriosclerotic; Binswanger's Disease; Binswangers Disease; Disease, Binswanger's; Encephalopathy, Subcortical, Chronic Progressive; Subcortical Encephalopathy, Chronic Progressive; Subcortical Leukoencephalopathy; Subcortical Arteriosclerotic Encephalopathy; Arteriosclerotic Encephalopathy, Subcortical; Arteriosclerotic Encephalopathies, Subcortical; Encephalopathies, Subcortical Arteriosclerotic; Subcortical Arteriosclerotic Encephalopathies; Encephalopathy, Binswanger's;

Binswanger's Encephalopathy; Encephalopathy, Binswangers; Encephalopathy, Binswanger; Encephalopathy, Chronic Progressive Subcortical; Subcortical ischemie vascular dementia; Vascular cognitive disorders; Vascular neurocognitive disorders; Vascular cognitive disorders; Vascular neurocognitive disorder; Vascular mild cognitive impairment; Vascular cognitive impairment, no dementia; Post stroke cognitive impairment; Post-stroke dementia; Subcortical vascular dementia; Cognitive impairment no dementia because of cerebrovascular disease; Ischemic vascular dementia; Subcortical vascular disease; Primary degenerative dementia; Mixed dementia; Cerebrovascular cognitive impairment; Multi infarct dementia; Subcortical ischemic vascular disease; Cognitive Dysfunctions; Dysfunction, Cognitive; Dysfunctions, Cognitive; Cognitive Impairments; Cognitive Impairment; Impairment, Cognitive; Impairments, Cognitive; Mild Cognitive Impairment; Cognitive Impairment, Mild; Cognitive Impairments, Mild; Impairment, Mild Cognitive; Impairments, Mild Cognitive; Mild Cognitive Impairments; Mild Neurocognitive Disorder; Disorder, Mild Neurocognitive; Disorders, Mild Neurocognitive; Mild Neurocognitive Disorders; Neurocognitive Disorder, Mild; Neurocognitive Disorders, Mild; Cognitive Decline; Cognitive Declines; Decline, Cognitive; Declines, Cognitive; Mental Deterioration; Deterioration, Mental; Deteriorations, Mental; Mental Deteriorations

②Chinese medicine treatment related:

1）Mesh of TCM：Chinese Medicine

Synonyms: Chinese Herbal Medicine; Medicine, Chinese Traditional; Traditional Chinese Medicine; Chinese Drug; Chinese Medicinal Formulas; Fang Ji Fen Lei; Fang-Ji-Fen-Lei; Fangji Fenlei; Fangji-Fenlei; Fangjifenlei; Traditional Chinese Medicinal Formula; Traditional Chinese Medicinal Formulas

2）Mesh of Traditional medicine：Traditional medicine

Synonyms： Ethnomedicine; Folk Medicine; Folk Remedy; Indigenous Medicine; Medicine, Traditional; Native Healing; Native Medicine; Traditional Healing; Traditional Indigenous Medicine

3）Mesh of Chinese herbal medicine：Herbal Medicine

Synonyms：Botanical Medicine; Herb Medicine; Medicine, Herbal; Medicine, Herbal; Phyto-Medicine; Phytomedicine; Plant Medicine; Plant-Based Medicine

4）Mesh of acupuncture：Acupuncture

Synonyms ： Acupuncture Therapy; Auriculotherapy; Point, Acupuncture; Acupuncture, Electric; Electric Acupuncture; Cupuncture, Ear; Acupuncture, Earlobe; Ear Acupuncture; Earlobe Acupuncture; Acupuncture Needle, Single-Use; Needle, Acupuncture; Acupuncture Anaesthesia; Acupuncture Anesthesia; Analgesia, Acupuncture

5）Mesh of cupping therapy：Cupping Therapy

Synonyms： Cupping (Therapy); Cupping Manipulation; Cupping Treatment; Fire Cupping; Flash Cupping; Moving Cupping; Suction Cupping; Vacuum Cupping

6）Mesh of Qigong：Qigong

Synonyms：Chi Kung; Chigung

7）Mesh of Tai Ji：Tai Chi

Synonyms：Tai Chi Chuan; Tai Ji; Taiji Quan; Taijiquan

8）TCM treatment in addition to the above words other words:

Complementary therapies; Therapies, Complementary; Therapy, Complementary; Complementary Medicine; Medicine, Complementary; Alternative Medicine; Medicine, Alternative; Alternative Therapies; Therapies, Alternative; Therapy, Alternative; Moxabustien; Massage; Zone Therapy; Therapies, Zone; Zone Therapies; Therapy, Zone; Massage Therapy; Massage Therapies; Therapies, Massage;Therapy, Massage; Baduanjin; Tuina

2.2 Each database specific retrieval strategy

2.2.1 CNKI

Professional search, the search time is from the establishment to March 1, 2021

(SU = ' Cognitive impairment ' OR SU = ' Impaired cognitive ' OR SU = ' Cognitive dysfunction ' OR SU = ' Cognitive impairment ' OR SU = ' Impairment of cognitive function ' OR SU = ' Mild cognitive impairment ' OR SU = ' Cognitive decline ' OR

SU = ' Cognitive impairment in diabetes ' OR SU = ' Amnestic mild cognitive impairment ' OR SU = ' Non-memory mild cognitive impairment ' OR SU = ' Cognitive mild cognitive impairment ' OR SU = ' Non-dementia cognitive impairment ' OR SU = ' Vascular cognitive impairment ' OR SU = ' Mild vascular cognitive impairment ' OR SU = ' Mild vascular cognitive dysfunction ' OR SU = ' Severe vascular cognitive impairment ' OR SU = ' Severe vascular cognitive dysfunction ' OR SU = ' Vascular dementia ' OR SU = ' Post-stroke dementia ' OR SU = ' Subcortical ischemic vascular dementia ' OR SU = ' Multiple infarcted dementia ' OR SU = ' Mixed dementia ' OR SU = ' Risk factors associated with vascular cognitive impairment ' OR SU = ' Ischemic vascular cognitive impairment ' OR SU = ' Hemorrhagic vascular cognitive impairment ' OR SU = ' Other cerebrovascular diseases of vascular cognitive impairment ' OR SU = ' Cerebrovascular disease with AD ' OR SU = ' Nondemented vascular cognitive impairment ' OR SU = ' Nondemented vascular cognitive dysfunction ' OR SU = ' Cognitive impairment after stroke ' OR SU = ' Cognitive impairment after stroke ' OR SU = ' Cognitive impairment in cerebrovascular disease ' OR SU = ' Cognitive dysfunction in lacunar infarction ' OR SU = ' Cognitive impairment after stroke ' OR SU = ' White matter osteoporosis cognitive impairment ' OR SU = ' Post-stroke dementia ' OR SU = ' Ischemic cognitive impairment ' OR SU = ' Multiple infarcted dementia ' OR SU = ' Small vascular dementia ' OR SU = ' Strategic site infarct dementia ' OR SU = 'Hypoperfusion Dementia 'OR SU =' Hemorrhagic Dementia 'OR SU = 'Autosomal Dominant Cerebral Artery Disease with Subcortical Infraction and Leukoencephalopathy 'OR SU =' Subcortical Vascular Cognitive Impair 'OR SU= 'Atherosclerotic cognitive impairment 'OR SU =' Moyamoya disease cognitive impairment 'OR SU = 'dementia 'OR SUOR SU = "not wise" OR SU = "amnesia" OR SU = "amnesia" OR SU = "brain extinction" OR SU = "text delusion" OR SU = "wrong language forgetfulness" OR SU = "ignorance" OR SU = "stupidity" OR SU=' stupid 'OR SU=' stupid' OR SU=' stupid 'OR SU=' idiot' OR SU=' Wai') AND (SU=' Chinese medicine 'OR SU=' Western medicine' OR SU=' Chinese medicine 'OR SU=' Chinese medicine' OR SU=' Chinese medicine ')OR SU= "Chinese patent medicine"

OR SU= "Chinese herbal medicine" OR SU= "herbal medicine" OR SU= "compound" OR SU= "acupuncture" OR SU= "acupuncture" OR SU= "warm needle" OR SU= "head needle" OR SU= "tongue needle" OR SU=' ear needle 'OR SU=' body needle' OR SU=' fire needle 'OR SU=' eye needle' OR SU=' electroacupuncture 'OR SU=' Plum flower needle' OR SU=' acupuncture 'OR SU=' scraping' OR SU=' cupping 'OR SU=' moxibustion' OR SU= "Massage" OR SU= "Qigong" OR SU= "Tai Chi" OR SU= "Baduanjin" OR SU= "Tie" OR SU= "Integrated Medicine" OR SU= "Complementary and Alternative Medicine" OR SU= "Alternative Medicine" OR SU= "Traditional Medicine" OR SU= "Su" OR SU = 'massage' OR SU = 'soup' OR SU = 'blood-letting' OR SU = 'blood-piercing' OR SU = 'tablet' OR SU = 'pill' OR SU = 'powder' OR SU = 'capsule' OR SU = 'mixture 'OR SU =' water 'OR SU = 'paste 'OR SU =' granule 'OR SU = 'injection 'OR SU = 'Dan 'OR SU = 'wine' OR SU = 'tea' OR SU = 'dew ')

A total of 12,602 articles were retrieved. There are 5999 papers in Chinese and 6603 papers in English. NE 5999 was actually imported and 5810 papers were rewritten.

2.2.2 WanFang

Professional search, the search time is from the establishment to March 1, 2021

Topic:(" cognitive impairment "OR" cognitive impairment "OR" cognitive impairment "OR" vascular cognitive impairment "OR" vascular dementia "OR" post-stroke dementia "OR" subcortical ischemic vascular dementia "OR" infarct dementia ")OR "mixed dementia" OR "ischemic vascular cognitive impairment" OR "hemorrhagic vascular cognitive impairment" OR "cerebrovascular disease with AD" OR" non-dementia vascular cognitive impairment "OR" non-dementia vascular cognitive impairment "OR" post-stroke cognitive impairment "OR "Small vascular disease cognitive impairment" OR "lacunar infarction cognitive dysfunction" OR "post-stroke cognitive impairment" OR "white matter osteoporosis cognitive impairment" OR "post-stroke dementia" OR "ischemic cognitive impairment" OR "small vascular dementia" OR "hypoperfusion dementia" OR "hemorrhagic dementia"

OR "Autosomal dominant cerebral artery disease of subcortical infarction and leukoencephalopathy" OR "moyamoya disease" cognitive impairment "OR" dementia "OR" forgetfulness "OR" amnesia "OR" amnesia "OR" brain myelopathy "OR" text delusion "OR" language amnesia "OR "Stupidity" OR "stupidity" OR "stupidity" OR "stupidity" OR "stupidity" OR "stupidity" OR "stupidity" OR "idiocy" OR "Wai") AND (" traditional Chinese medicine "OR" traditional Chinese AND western medicine "OR" patent Chinese medicine "OR" herbal medicine "OR" prescription "OR" compound "OR" acupuncture "OR "Acupuncture" OR "warm acupuncture" OR "head acupuncture" OR "ear acupuncture" OR "fire acupuncture" OR "plum flower acupuncture" OR "acupoint" OR "scraping" OR "cupping" OR "moxibustion" OR "massage" OR "Qigong" OR "Tai Chi" OR "Baduanjin" OR "Paste" OR "integrated medicine" OR "alternative medicine" OR "traditional medicine" OR "massage" OR "soup" OR "blood-letting" OR "blood-pricking" OR "tablet" OR "pill" OR "powder" OR "capsule" OR "granule" OR "liquid" OR "Agent" OR "cream" OR "of" OR "injection" OR "Dan" OR "wine" OR "tea" OR "dew"))

A total of 4672 articles were searched. There are 4464 papers in Chinese and 208 in English, 4464 papers in NE were actually imported and 4413 papers were rewritten.

2.2.3 VIP

The retrieval time is from the establishment of the database to March 1, 2021

(M= Cognitive Impairment OR Cognitive Impairment OR Cognitive Impairment OR Cognitive Impairment OR Mild OR Decline OR Diabetic OR Amnesiac Mild OR Non-Memory Mild OR Cognitive Mild OR non-dementia OR mild OR mild vascular cognitive impairment OR severe OR severe vascular dementia OR post-stroke dementia OR subcortical ischemic vascular dementia OR Multiple infarct dementia OR mixed dementia OR risk factor-related vascular cognitive impairment OR ischemic vascular cognitive impairment OR hemorrhagic vascular cognitive impairment OR other cerebrovascular vascular cognitive impairment OR cerebrovascular disease with AD OR non-dementia vascular cognitive impairment

OR Non-dementia vascular cognitive impairment OR post-stroke cognitive impairment OR small vascular disease OR lacunar infarction cognitive impairment OR post-stroke cognitive impairment OR white matter osteoporosis cognitive impairment OR post-stroke dementia OR ischemic cognitive impairment OR multiple infarct dementia OR small vascular dementia OR strategic site infarction dementia OR hypoperfusion dementia OR hemorrhagic dementia OR autosomal dominant cerebral artery disease with subcortical infarction and leukoencephalopathy OR "subcortical vascular cognitive impairment (SVCI)" OR large atherosclerotic cognitive impairment OR moyamoya disease cognitive impairment OR dull disease OR dull syndrome OR forgetfulness OR forgetfulness OR idiocy OR not OR " Wai "OR dementia OR stupid OR crazy OR stupid OR stupid OR god stupid OR happy to forget OR brain marrow disappear OR text OR language wrong forget) AND (M= Chinese AND western medicine OR Chinese medicine OR traditional Chinese medicine OR Chinese medicine OR herbal medicine OR herbal medicine OR compound OR acupuncture OR acupuncture OR head acupuncture OR ear acupuncture OR body acupuncture OR electroacupuncture OR plum flower acupuncture OR needle OR blood-letting OR pricking OR acupuncture OR acupuncture OR scraping OR massage OR cupping OR moxibustion OR massage OR Qigong OR Tai Chi OR Ba Duan broam OR paste OR medicine bath OR foot bath OR acupoint injection OR combined medicine OR complementary and alternative medicine OR alternative therapy OR traditional medicine OR ethnic medicine OR soup OR tablet OR pill Powder OR capsule OR granule OR liquid OR mixture OR water OR cream OR infusion OR injection OR wine OR tea OR dew

A total of 5200 articles were searched. 5172 Chinese papers, 28 English papers, 5200 NE papers were actually imported, and 4790 papers were removed.

2.2.4 SinoMed

Advanced search, the search time is from the database establishment to March 1, 2021

#1 (" Cognitive impairment "[Frequent Field: Intelligence] OR "Cognitive impairment "[Frequent Field: Intelligence] OR" Cognitive impairment "[Frequent Field:

Intelligence] OR "Cognitive impairment "[Frequent Field: Intelligence] OR" Cognitive impairment "[Frequent Field: Intelligence] OR "Cognitive impairment" "Mild Cognitive Impairment "[Frequent Field: Intelligence] OR" Cognitive Decline "[Frequent Field: Intelligence] OR "Diabetic Cognitive Impairment "[Frequent Field: Intelligence] OR" Amnesia Mild Cognitive Impairment "[Frequent Field: Intelligence]) AND -2021[Date] 28972

#2 "Non-memory Mild Cognitive Impairment "[Frequent Field: Intelligence] OR" Cognitive Mild Cognitive Impairment "[Frequent Field: Intelligence] OR "Cognitive Impairment Without Dementia "[Frequent Field: Intelligence] OR" Vascular Cognitive Impairment "[Frequent Field: Intelligence] OR "Mild Vascular Cognitive Impairment "[Frequent Field: Intelligence] OR" Vascular Cognitive Impairment" "Mild Vascular Cognitive Impairment "[Frequent Field: Intelligence] OR" Severe Vascular Cognitive Impairment "[Frequent Field: Intelligence] OR "Severe Vascular Dementia "[Frequent Field: Intelligence]11922

#3 "Post-stroke dementia "[Commonly used fields: intelligence] OR" Subcortical ischemic vascular dementia "[Commonly used fields: intelligence] OR "Multiple infarct dementia "[Commonly used fields: intelligence] OR" Mixed dementia "[Commonly used fields: intelligence] OR "Risk factor-related vascular cognitive impairment "[Commonly used fields: intelligence] OR "Ischemic Vascular Cognitive Impairment "[Commonly used Field: Intelligence] OR" Hemorrhagic Vascular Cognitive Impairment "[Commonly used Field: Intelligence] OR "Other Vascular Cognitive Impairment of Cerebrovascular Disease "[Commonly used Field: Intelligence] OR" Cerebrovascular Disease with AD"[Commonly used Field: Intelligence]317

#4 "Non-Dementia Vascular Cognitive Disorder "[Frequent Field: Intelligence] OR" Non-Dementia Vascular Cognitive Disorder "[Frequent Field: Intelligence] OR "Post-stroke Cognitive Disorder "[Frequent Field: Intelligence] OR" Post-stroke Cognitive Disorder "[Frequent Field: Intelligence] OR "Small cerebral vascular disease cognitive impairment "[commonly used fields: intelligence] OR" lacunar infarction cognitive dysfunction "[commonly used fields: intelligence] OR

"post-stroke cognitive impairment "[commonly used fields: intelligence] OR" white matter osteoporosis cognitive impairment "[commonly used fields: intelligence] OR "post-stroke dementia "[commonly used fields: intelligence] 546

#5 "Ischemic Cognitive Impairment "[Commonly used Field: Intelligence] OR" Multiple Infarcted Dementia "[Commonly used Field: Intelligence] OR "Small Vascular Dementia "[Commonly used Field: Intelligence] OR" Strategic Site Infarcted Dementia "[Commonly used Field: Intelligence] OR "Low Perfusion Dementia "[Commonly used Field: Intelligence] OR "Hemorrhagic dementia "[Commonly used fields: intelligence] OR" autosomal dominant cerebral artery disease with subcortical infarction and leukinencephalopathy "[Commonly used fields: intelligence] OR "subcortical vascular cognitive impairment (SVCI) "[Commonly used fields: intelligence] OR" aortic atherosclerotic cognitive impairment "[Commonly used fields: intelligence]96

6 "stay delusion" [common fields: intelligent] OR "chi card" [common fields: intelligent] OR "ignorance" [common fields: intelligent] OR "god" [common fields: intelligent] OR "xi forget [common fields: intelligent]" OR "brain subsides" [common fields: intelligent] OR "wen chi" [common fields: intelligent] OR "Language Mistake "[Commonly used field: intelligence]162

#7 "Moyamoya disease cognitive impairment "[Commonly used field: intelligence] OR" stupidity "[Commonly used field: intelligence] OR "forgetfulness "[Commonly used field: intelligence] OR" forgetfulness "[Commonly used field: intelligence] OR "idiocy "[Commonly used field: intelligence] OR "Not hui "[commonly used field: intelligence] OR" dementia "[commonly used field: intelligence] OR " 纇 (wai) "[commonly used field: intelligence]56127

#8 (#7) OR (#6) OR (#5) OR (#4) OR (#3) OR (#2) OR (#1)76793

9 (" water "[common fields: intelligent] OR" paste "[common fields: intelligent] OR" infusion of [common fields: intelligent] "OR" injection "[common fields: intelligent] OR" injection "[common fields: intelligent] OR" Dan "[common fields: intelligent] OR" wine "[common fields: intelligent] the OR "Tea" [common fields: intelligent] OR "dew" [common fields: intelligent]) OR (" national medicine

[common fields: intelligent] "OR" soup "[common fields: intelligent] OR" slices "[common fields: intelligent] OR" pill "[common fields: intelligent] OR" loose "[common fields: intelligent] the OR "Capsule" [common fields: intelligent] OR "particles" [common fields: intelligent] OR "fluid" [common fields: intelligent] OR "agent" [common fields: intelligent]) OR (" eight brocade "[common fields: intelligent] OR" posted "[common fields: intelligent] OR" dipping "[common fields: intelligent] the OR "Foot Bath "[Frequent Field: Intelligence] OR" Acupoint Injection "[Frequent Field: Intelligence] OR "Integrated Medicine "[Frequent Field: Intelligence] OR" Complementary and Alternative Medicine "[Frequent Field: Intelligence] OR "Alternative Medicine "[Frequent Field: Intelligence] OR" Traditional Medicine "[Frequent Field: Intelligence]) OR (" stab winding "[common fields: intelligent] OR" hole "[common fields: intelligent] OR" scrapping "[common fields: intelligent] OR" massage "[common fields: intelligent] OR" cupping "[common fields: intelligent] OR" moxibustion "[common fields: intelligent] OR" massage "[common fields: intelligent] the OR "Qigong" [common fields: intelligent] OR "tai chi" [common fields: intelligent]) OR (" acupuncture "[common fields: intelligent] OR" pin "[common fields: intelligent] OR" earpins [common fields: intelligent] "OR" body acupuncture "[common fields: intelligent] OR" cupping "[common fields: intelligent] the OR "Plum needle [common fields: intelligent]" OR "needle" [common fields: intelligent] OR "bleeding" [common fields: intelligent] OR "blood" [common fields: intelligent]) OR (" traditional Chinese "[common fields: intelligent] OR" TCM "[common fields: intelligent] OR" Chinese medicine "[common fields: intelligent] OR "Chinese patent medicine "[Commonly used field: intelligence] OR" Chinese herbal medicine "[Commonly used field: intelligence] OR "herbal medicine "[Commonly used field: intelligence] OR" compound "[Commonly used field: intelligence] OR "acupuncture "[Commonly used field: intelligence]) 5131622

#10 (#9) AND (#8) 38834

A total of 38834 articles were retrieved. Chinese 38676, English 158, the actual import of NE 38834, reduplication of 36346.

2.2.5 Pubmed

Retrieval time: until March 1, 2021

#1 "Cognitive Dysfunction"[MeSH Terms] **18,312**

#2 ((((((((((((((((((((((((((((Cognitive Dysfunction[Title/Abstract]) OR (Cognitive Dysfunctions[Title/Abstract])) OR (Dysfunction, Cognitive[Title/Abstract])) OR (Dysfunctions, Cognitive[Title/Abstract])) OR (Cognitive Impairments[Title/Abstract])) OR (Cognitive Impairment[Title/Abstract])) OR (Impairment, Cognitive[Title/Abstract])) OR (Impairments, Cognitive[Title/Abstract])) OR (Mild Cognitive Impairment[Title/Abstract])) OR (Cognitive Impairment, Mild[Title/Abstract])) OR (Cognitive Impairments, Mild[Title/Abstract])) OR (Impairment, Mild Cognitive[Title/Abstract])) OR (Impairments, Mild Cognitive[Title/Abstract])) OR (Mild Cognitive Impairments[Title/Abstract])) OR (Mild Neurocognitive Disorder[Title/Abstract])) OR (Disorder, Mild Neurocognitive[Title/Abstract])) OR (Disorders, Mild Neurocognitive[Title/Abstract])) OR (Mild Neurocognitive Disorders[Title/Abstract])) OR (Neurocognitive Disorder, Mild[Title/Abstract]))) OR (Neurocognitive Disorders[Title/Abstract])) OR (Mild Cognitive Decline[Title/Abstract])) OR (Cognitive Declines[Title/Abstract])) OR (Decline, Cognitive[Title/Abstract])) OR (Declines, Cognitive[Title/Abstract])) OR (Mental Deterioration[Title/Abstract])) OR (Deterioration, Mental[Title/Abstract])) OR (Deteriorations, Mental[Title/Abstract])) OR (Mental Deteriorations[Title/Abstract]) **109,650**

#3 #1 OR #2 112,981

#4 Dementia, Vascular[MeSH Terms] **6,669**

#5 (((((((((((((((((((((((((((((((((((((((Dementia, Vascular[Title/Abstract]) OR (Dementias, Vascular[Title/Abstract])) OR (Vascular Dementias[Title/Abstract])) OR (Vascular Dementia[Title/Abstract])) OR (Vascular Dementia, Acute Onset[Title/Abstract])) OR (Acute Onset Vascular Dementia[Title/Abstract])) OR (Subcortical Vascular Dementia[Title/Abstract])) OR (Dementia, Subcortical

Vascular[Title/Abstract])) OR (Dementias, Subcortical Vascular[Title/Abstract])) OR (Subcortical Vascular Dementias[Title/Abstract])) OR (Vascular Dementia, Subcortical[Title/Abstract])) OR (Vascular Dementias, Subcortical[Title/Abstract])) OR (Arteriosclerotic Dementia[Title/Abstract])) OR (Arteriosclerotic Dementias[Title/Abstract])) OR (Dementia, Arteriosclerotic[Title/Abstract])) OR (Dementias, Arteriosclerotic[Title/Abstract])) OR (Binswanger Disease[Title/Abstract])) OR (Disease, Binswanger[Title/Abstract])) OR (Chronic Progressive Subcortical Encephalopathy[Title/Abstract])) OR (Binswanger Encephalopathy[Title/Abstract])) OR (Leukoencephalopathy, Subcortical[Title/Abstract])) OR (Leukoencephalopathies, Subcortical[Title/Abstract])) OR (Subcortical Leukoencephalopathies[Title/Abstract])) OR (Encephalopathy, Subcortical Arteriosclerotic[Title/Abstract])) OR (Binswanger's Disease[Title/Abstract])) OR (Binswangers Disease[Title/Abstract])) OR (Disease, Binswanger's[Title/Abstract])) OR (Encephalopathy, Subcortical, Chronic Progressive[Title/Abstract])) OR (Subcortical Encephalopathy, Chronic Progressive[Title/Abstract])) OR (Subcortical Leukoencephalopathy[Title/Abstract])) OR (Subcortical Arteriosclerotic Encephalopathy[Title/Abstract])) OR (Arteriosclerotic Encephalopathy, Subcortical[Title/Abstract])) OR (Arteriosclerotic Encephalopathies, Subcortical[Title/Abstract])) OR (Encephalopathies, Subcortical Arteriosclerotic[Title/Abstract])) OR (Subcortical Arteriosclerotic Encephalopathies[Title/Abstract])) OR (Encephalopathy, Binswanger's[Title/Abstract])) OR (Binswanger's Encephalopathy[Title/Abstract])) OR (Encephalopathy, Binswangers[Title/Abstract])) OR (Encephalopathy, Binswanger[Title/Abstract])) OR (Encephalopathy, Chronic Progressive Subcortical[Title/Abstract]) **9,058**

#6 #4 OR #5 **11,425**

#7 Dementia, Multi-Infarct[MeSH Terms] **1,089**

#8 ((((((((((((((((Dementia, Multi-Infarct[Title/Abstract]) OR (Dementia, Multi Infarct[Title/Abstract])) OR (Dementias, Multi-Infarct[Title/Abstract])) OR (Multi-Infarct Dementias[Title/Abstract])) OR (Dementia Multi-Infarct[Title/Abstract])) OR (Dementia Multi Infarct[Title/Abstract])) OR (Dementia Multi-Infarcts[Title/Abstract])) OR (Multi-Infarct, Dementia[Title/Abstract])) OR (Multi-Infarcts, Dementia[Title/Abstract])) OR (Dementia, Multiinfarct[Title/Abstract])) OR (Dementias, Multiinfarct[Title/Abstract])) OR (Multiinfarct Dementia[Title/Abstract])) OR (Multiinfarct Dementias[Title/Abstract])) OR (Multi-Infarct Dementia[Title/Abstract])) OR (Multi Infarct Dementia[Title/Abstract])) OR (Lacunar Dementia[Title/Abstract])) OR (Dementia, Lacunar[Title/Abstract])) OR (Dementias, Lacunar[Title/Abstract])) OR (Lacunar Dementias[Title/Abstract]) **1,810**

#9 #7 OR # 8 **2,414**

#10 (((((((((((((((Vascular Cognitive Impairment[Title/Abstract]) Or (Vascular Cognitive Disorders[Title/Abstract])) Or (Vascular Neurocognitive Disorders[Title/Abstract])) Or (Vascular Cognitive Disorders[Title/Abstract])) Or (Vascular Neurocognitive Disorder[Title/Abstract])) Or (Vascular Mild Cognitive Impairment[Title/Abstract])) Or (Vascular Cognitive Impairment No Dementia[Title/Abstract])) Or (Post Stroke Cognitive Impairment[Title/Abstract])) Or (Post Stroke Dementia[Title/Abstract])) Or (Subcortical Vascular Dementia[Title/Abstract])) Or (Cognitive Impairment No Dementia Because Of Cerebrovascular Disease[Title/Abstract])) Or (Ischemic Vascular Dementia[Title/Abstract])) Or (Subcortical Vascular Disease[Title/Abstract])) Or (Primary Degenerative Dementia[Title/Abstract])) Or (Mixed Dementia[Title/Abstract])) Or (Cerebrovascular Cognitive Impairment[Title/Abstract])) Or (Subcortical Ischemic Vascular Disease[Title/Abstract]) **5,218**

#11 #3 OR #6 OR #9 OR #10 **122,076**

#12 (((((((((((((Medicine, Chinese Traditional[Mesh Terms]) Or (Medicine, Chinese Traditional[Title/Abstract])) Or (((((((((((((((Traditional Chinese Medicine[Title/Abstract]) Or (Chung I Hsueh[Title/Abstract])) Or (Hsueh, Chung I[Title/Abstract])) Or (Traditional Medicine, Chinese[Title/Abstract])) Or (Zhong Yi Xue[Title/Abstract])) Or (Chinese Traditional Medicine[Title/Abstract])) Or (Chinese Medicine, Traditional[Title/Abstract])) Or (Traditional Tongue Diagnosis[Title/Abstract])) Or (Tongue Diagnoses, Traditional[Title/Abstract])) Or (Tongue Diagnosis, Traditional[Title/Abstract])) Or (Traditional Tongue Diagnoses[Title/Abstract])) Or (Traditional Tongue Assessment[Title/Abstract])) Or (Tongue Assessment, Traditional[Title/Abstract])) Or (Traditional Tongue Assessments[Title/Abstract])) Or (Medicine, Chinese Traditional[Title/Abstract]))) Or (((Herbal Medicine[Mesh Terms]) Or (((((((((((Medicine, Herbal[Title/Abstract]) Or (Hawaiian Herbal Medicine[Title/Abstract])) Or (Hawaiian Herbal Medicines[Title/Abstract])) Or (Herbal Medicine, Hawaiian[Title/Abstract])) Or (Herbal Medicines, Hawaiian[Title/Abstract])) Or (Medicine, Hawaiian Herbal[Title/Abstract])) Or (Medicines, Hawaiian Herbal[Title/Abstract])) Or (La'Au Lapa'Au[Title/Abstract])) Or (Laau Lapaau[Title/Abstract])) Or (La Au Lapa Au[Title/Abstract])) Or (Herbalism[Title/Abstract]))) Or (Herbal Medicine))) Or (((Complementary Therapies[Mesh Terms]) Or (Complementary Therapies[Title/Abstract])) Or (((((((((Therapies, Complementary[Title/Abstract]) Or (Therapy, Complementary[Title/Abstract])) Or (Complementary Medicine[Title/Abstract])) Or (Medicine, Complementary[Title/Abstract])) Or (Alternative Medicine[Title/Abstract])) Or (Medicine, Alternative[Title/Abstract])) Or (Alternative Therapies[Title/Abstract])) Or (Therapies, Alternative[Title/Abstract])) Or (Therapy, Alternative[Title/Abstract])))) Or ((Acupuncture[Mesh Terms]) Or ((((((((((((((((((((((((((((((Acupuncture[Title/Abstract]) Or (Acupuncture Therapy[Title/Abstract])) Or (Point, Acupuncture[Title/Abstract])) Or

(Pharmacopuncture[Title/Abstract])) Or (Acupuncture Treatment[Title/Abstract])) Or (Acupuncture Treatments[Title/Abstract])) Or (Treatment, Acupuncture[Title/Abstract])) Or (Therapy, Acupuncture[Title/Abstract])) Or (Pharmaceacupuncture[Title/Abstract])) Or (Treatment, Pharmacoacupuncture[Title/Abstract])) Or (Pharmacoacupuncture Therapy[Title/Abstract])) Or (Therapy;Pharmacacupunsture[Title/Abstract])) Or (Acup9tomy[Title/Abstract])) Or (Asuretomies[Title/Abstract])) Or (Acupunctures, Ear[Title/Abstract])) Or (Ear Acupunctures[Title/Abstract])) Or (Auricular Acupuncture[Title/Abstract])) Or (Ear Acupuncture[Title/Abstract])) Or (Acupuncture, Auricular[Title/Abstract])) Or (Acupunctures, Auricular[Title/Abstract])) Or (Auricular Acupunctures[Title/Abstract])) Or (Acupuncture Point[Title/Abstract])) Or (Point, Acupuncture[Title/Abstract])) Or (Points, Acupuncture[Title/Abstract])) Or (Acupoints[Title/Abstract])) Or (Acupoint[Title/Abstract])) Or (Analgesia[Title/Abstract])) Or (Acupuncture[Title/Abstract])) Or (Acupuncture Anesthesia[Title/Abstract])) Or (Anesthesia, Acupuncturee[Title/Abstract])))) Or ((Moxibustion[Mesh Terms]) Or (Moxibustion[Title/Abstract]))) Or ((Massage[Mesh Terms]) Or ((((((((Massage[Title/Abstract]) Or (Zone Therapy[Title/Abstract])) Or (Therapies, Zone[Title/Abstract])) Or (Zone Therapies[Title/Abstract])) Or (Therapy, Zone[Title/Abstract])) Or (Massage Therapy[Title/Abstract])) Or (Massage Therapies[Title/Abstract])) Or (Therapies, Massage[Title/Abstract])) Or (Therapy, Massage[Title/Abstract])))) Or ((Cupping Therapy[Mesh Terms]) Or ((((((Cupping Therapy[Title/Abstract]) Or (Cupping Therapies[Title/Abstract])) Or (Therapy, Cupping[Title/Abstract])) Or (Cupping Treatment[Title/Abstract])) Or (Cupping Treatments[Title/Abstract])) Or (Treatment, Cupping[Title/Abstract])))) Or ((Qigong[Mesh Terms]) Or (((Qigong[Title/Abstract]) Or (Qi Gong[Title/Abstract])) Or (Ch'i Kung[Title/Abstract])))) Or ((Tai Ji[Mesh Terms]) Or (((((((((Tai Ji[Title/Abstract]) Or (Taichi[Title/Abstract])) Or (Chi, Tai[Title/Abstract])) Or (Tai Ji Quan[Title/Abstract])) Or (Ji Quan,Tai[Title/Abstract])) Or (Quan,Tai

Ji[Title/Abstract])) Or (Taiji[Title/Abstract])) Or (Taijiquan[Title/Abstract])) Or (Tlai Chi[Title/Abstract])) Or (Tai Chi Chuan[Title/Abstract])))) Or (Baduanjin[Title/Abstract])) Or (Tuina[Title/Abstract])) 386,900

#13 #11 AND #12 **2,438**

A total of 2438 articles were retrieved.NE 2438 is actually imported, and the last 2432 is removed.

2.2.6 Cochrane library

Retrieval time: until March 1, 2021

#1 MeSH descriptor: [Dementia, Vascular] explode all trees

#2 (Vascular Dementias):ti,ab,kw OR (Dementias, Vascular):ti,ab,kw OR (Vascular Dementia):ti,ab,kw OR (Dementia, Subcortical Vascular):ti,ab,kw OR (Vascular Dementia, Subcortical):ti,ab,kw OR (Dementias, Subcortical Vascular):ti,ab,kw OR (Subcortical Vascular Dementias):ti,ab,kw OR (Vascular Dementias, Subcortical):ti,ab,kw OR (Subcortical Vascular Dementia):ti,ab,kw OR (Binswanger's Encephalopathy):ti,ab,kw OR (Binswanger Encephalopathy):ti,ab,kw OR (Chronic Progressive Subcortical Encephalopathy):ti,ab,kw OR (Encephalopathy, Binswangers):ti,ab,kw OR (Arteriosclerotic Encephalopathies, Subcortical):ti,ab,kw OR (Disease, Binswanger):ti,ab,kw OR (Arteriosclerotic Encephalopathy, Subcortical):ti,ab,kw OR (Encephalopathies, Subcortical Arteriosclerotic):ti,ab,kw OR (Subcortical Arteriosclerotic Encephalopathies):ti,ab,kw OR (Subcortical Arteriosclerotic Encephalopathy):ti,ab,kw OR (Encephalopathy, Subcortical, Chronic Progressive):ti,ab,kw OR (Leukoencephalopathies, Subcortical):ti,ab,kw OR (Subcortical Leukoencephalopathies):ti,ab,kw OR (Encephalopathy, Binswanger's):ti,ab,kw OR (Encephalopathy, Chronic Progressive Subcortical):ti,ab,kw OR (Disease, Binswanger's):ti,ab,kw OR (Encephalopathy, Subcortical Arteriosclerotic):ti,ab,kw OR (Leukoencephalopathy, Subcortical):ti,ab,kw OR (Subcortical Leukoencephalopathy):ti,ab,kw OR

(Binswangers Disease):ti,ab,kw OR (Subcortical Encephalopathy, Chronic Progressive):ti,ab,kw OR (Binswanger's Disease):ti,ab,kw OR (Binswanger Disease):ti,ab,kw OR (Encephalopathy, Binswanger):ti,ab,kw OR (Acute Onset Vascular Dementia):ti,ab,kw OR (Vascular Dementia, Acute Onset):ti,ab,kw OR (Arteriosclerotic Dementias):ti,ab,kw OR (Dementia, Arteriosclerotic):ti,ab,kw OR (Arteriosclerotic Dementia):ti,ab,kw OR (Dementias, Arteriosclerotic):ti,ab,kw

#3 #1 OR #2

#4 MeSH descriptor: [Dementia, Multi-Infarct] explode all trees

#5 (Multi-Infarct Dementias):ti,ab,kw OR (Multi Infarct Dementia):ti,ab,kw OR (Multi-Infarct, Dementia):ti,ab,kw OR (Multiinfarct Dementia):ti,ab,kw OR (Multi-Infarct Dementia):ti,ab,kw OR (Dementia Multi-Infarcts):ti,ab,kw OR (Dementia, Multi Infarct):ti,ab,kw OR (Dementias, Multi-Infarct):ti,ab,kw OR (Multi-Infarcts, Dementia):ti,ab,kw OR (Dementia, Multiinfarct):ti,ab,kw OR (Dementia Multi-Infarct):ti,ab,kw OR (Dementia Multi Infarct):ti,ab,kw OR (Dementias, Multiinfarct):ti,ab,kw OR (Multiinfarct Dementias):ti,ab,kw OR (Dementia, Lacunar):ti,ab,kw OR (Dementias, Lacunar):ti,ab,kw OR (Lacunar Dementia):ti,ab,kw OR (Lacunar Dementias):ti,ab,kw

#6 #4 OR #5

#7 MeSH descriptor: [Cognitive Dysfunction] explode all trees

#8 (Dysfunctions, Cognitive):ti,ab,kw OR (Cognitive Impairments):ti,ab,kw OR (Impairments, Cognitive):ti,ab,kw OR (Impairment, Cognitive):ti,ab,kw OR (Dysfunction, Cognitive):ti,ab,kw OR (Cognitive Dysfunctions):ti,ab,kw OR (Cognitive Impairment):ti,ab,kw OR (Mild Neurocognitive Disorders):ti,ab,kw OR (Impairment, Mild Cognitive):ti,ab,kw OR (Neurocognitive Disorders, Mild):ti,ab,kw OR (Mild Cognitive Impairment):ti,ab,kw OR (Impairments, Mild Cognitive):ti,ab,kw OR (Disorder, Mild Neurocognitive):ti,ab,kw OR (Mild Cognitive Impairments):ti,ab,kw OR (Cognitive Impairment, Mild):ti,ab,kw OR (Mild Neurocognitive Disorder):ti,ab,kw OR (Disorders, Mild Neurocognitive):ti,ab,kw OR (Neurocognitive Disorder, Mild):ti,ab,kw OR

(Cognitive Impairments, Mild):ti,ab,kw OR (Mental Deteriorations):ti,ab,kw OR (Decline, Cognitive):ti,ab,kw OR (Cognitive Decline):ti,ab,kw OR (Mental Deterioration):ti,ab,kw OR (Deteriorations, Mental):ti,ab,kw OR (Deterioration, Mental):ti,ab,kw OR (Declines, Cognitive):ti,ab,kw OR (Cognitive Declines):ti,ab,kw

#9 #7 OR #8

#10 (Vascular Cognitive Impairment):ti,ab,kw OR (Vascular Cognitive Disorders):ti,ab,kw OR (Vascular Neurocognitive Disorders):ti,ab,kw OR (Vascular Cognitive Disorders):ti,ab,kw OR (Vascular Neurocognitive Disorder):ti,ab,kw OR (Vascular Mild Cognitive Impairment):ti,ab,kw OR (Vascular Cognitive Impairment No Dementia):ti,ab,kw OR (Post Stroke Cognitive Impairment):ti,ab,kw OR (Post Stroke Dementia):ti,ab,kw OR (Subcortical Vascular Dementia):ti,ab,kw OR (Cognitive Impairment No Dementia Because Of Cerebrovascular Disease):ti,ab,kw OR (Ischemic Vascular Dementia):ti,ab,kw OR (Subcortical Vascular Disease):ti,ab,kw OR (Primary Degenerative Dementia):ti,ab,kw OR (Mixed Dementia):ti,ab,kw OR (Cerebrovascular Cognitive Impairment):ti,ab,kw OR (Subcortical Ischemic Vascular Disease):ti,ab,kw

#11 #3 OR #6 OR #9 OR #10

#12 MeSH descriptor: [Medicine, Chinese Traditional] explode all trees

#13 (Traditional Medicine, Chinese):ti,ab,kw OR (Chinese Traditional Medicine):ti,ab,kw OR (Zhong Yi Xue):ti,ab,kw OR (Chung I Hsueh):ti,ab,kw OR (Hsueh, Chung I):ti,ab,kw OR (Chinese Medicine, Traditional):ti,ab,kw OR (Traditional Chinese Medicine):ti,ab,kw OR (Tongue Diagnosis, Traditional):ti,ab,kw OR (Traditional Tongue Assessments):ti,ab,kw OR (Traditional Tongue Diagnoses):ti,ab,kw OR (Traditional Tongue Assessment):ti,ab,kw OR (Tongue Assessment, Traditional):ti,ab,kw OR (Tongue Diagnoses, Traditional):ti,ab,kw OR (Traditional Tongue Diagnosis):ti,ab,kw

#14 #12 OR #13

#15 MeSH descriptor: [Herbal Medicine] explode all trees

#16 (Medicine, Herbal):ti,ab,kw OR (La au Lapa au):ti,ab,kw OR (La'au Lapa'au):ti,ab,kw OR (Herbal Medicine, Hawaiian):ti,ab,kw OR (Herbal Medicines, Hawaiian):ti,ab,kw OR (Laau Lapaau):ti,ab,kw OR (Hawaiian Herbal Medicine):ti,ab,kw OR (Hawaiian Herbal Medicines):ti,ab,kw OR (Medicines, Hawaiian Herbal):ti,ab,kw OR (Medicine, Hawaiian Herbal):ti,ab,kw OR (Herbalism):ti,ab,kw

#17 #15 OR #16

#18 MeSH descriptor: [Medicine, Traditional] explode all trees

#19 (Medicine, Primitive):ti,ab,kw OR (Primitive Medicine):ti,ab,kw OR (Remedies, Home;Remedy, Home):ti,ab,kw OR (Home Remedies):ti,ab,kw OR (Home Remedy):ti,ab,kw OR (Remedy, Folk):ti,ab,kw OR (Remedies, Folk):ti,ab,kw OR (Folk Remedy):ti,ab,kw OR (Folk Remedies):ti,ab,kw OR (Ethnomedicine):ti,ab,kw OR (Indigenous Medicine):ti,ab,kw OR (Medicine, Indigenous):ti,ab,kw OR (Medicine, Folk):ti,ab,kw OR (Folk Medicine):ti,ab,kw OR (Traditional Medicine):ti,ab,kw

#20 (ComplementaryTherapies):ti,ab,kw OR (Therapies, Complementary):ti,ab,kw OR (Therapy, Complementary):ti,ab,kw OR (Complementary Medicine):ti,ab,kw OR (Medicine, Complementary):ti,ab,kw OR (Alternative Medicine):ti,ab,kw OR (Medicine, Alternative):ti,ab,kw OR (Alternative Therapies):ti,ab,kw OR (Therapies, Alternative):ti,ab,kw OR (Therapy, Alternative):ti,ab,kw

#21 #18 OR #19

#22 MeSH descriptor: [Acupuncture] explode all trees

#23 (Pharmacopuncture):ti,ab,kw OR (Pharmacopunsture):ti,ab,kw OR (Acupuncture Treatment):ti,ab,kw OR (Acupuncture Treatments):ti,ab,kw OR (Treatment, Acupuncture):ti,ab,kw OR (Therapy Acupuncture):ti,ab,kw OR (Pharmaceacupuncture Treatment):ti,ab,kw OR (Treatment, Pharmacoacupuncture):ti,ab,kw OR (Pharmacoacupuncture Therapy):ti,ab,kw OR (Pharmacacupunsture):ti,ab,kw OR (Acupotomy):ti,ab,kw OR (AsuRetomies):ti,ab,kw OR (Acupunctures, Ear):ti,ab,kw OR (Ear

Acupunctures):ti,ab,kw OR (Auricular Acupuncture):ti,ab,kw OR (Ear Acupuncture):ti,ab,kw OR (Acupuncture, Auricular):ti,ab,kw OR (Acupunctures,Auricular):ti,ab,kw OR (Auricular Acupunctures):ti,ab,kw OR (Acupuncture Point):ti,ab,kw OR (Point,Acupuncture):ti,ab,kw OR (Points, Acupuncture):ti,ab,kw OR (Acupoints):ti,ab,kw OR (Acupoint):ti,ab,kw OR (Analgesia, Acupuncture):ti,ab,kw OR (AcupunctureAnesthesia):ti,ab,kw OR (Anesthesia,Acupuncturee):ti,ab,kw

#24 #22 OR #23

#25 MeSH descriptor: [Moxibustion] explode all trees

#26 (Moxabustion):ti,ab,kw

#27 #25 OR #26

#28 MeSH descriptor: [Massage] explode all trees

#29 (Zone Therapies):ti,ab,kw OR (Zone Therapy):ti,ab,kw OR (Therapies, Zone):ti,ab,kw OR (Therapy, Zone):ti,ab,kw OR (Massage Therapies):ti,ab,kw OR (Massage Therapy):ti,ab,kw OR (Therapy, Massage):ti,ab,kw OR (Therapies, Massage):ti,ab,kw

#30 #28 OR #29

#31 MeSH descriptor: [Cupping Therapy] explode all trees

#32 (Cupping Treatment):ti,ab,kw OR (Treatment, Cupping):ti,ab,kw OR (Cupping Treatments):ti,ab,kw OR (Cupping Therapies):ti,ab,kw OR (Therapy, Cupping):ti,ab,kw

#33 #31 OR #32

#34 MeSH descriptor: [Qigong] explode all trees

#35 (Ch i Kung):ti,ab,kw OR (Qi Gong):ti,ab,kw

#36 #34 OR #35

#37 MeSH descriptor: [Tai Ji] explode all trees

#38 (Tai Chi):ti,ab,kw OR (Taijiquan):ti,ab,kw OR (Tai Ji Quan):ti,ab,kw OR (Taiji):ti,ab,kw OR (Ji Quan, Tai):ti,ab,kw OR (Chi, Tai):ti,ab,kw OR (Tai Chi Chuan):ti,ab,kw OR (Quan, Tai Ji):ti,ab,kw OR (Tai-ji):ti,ab,kw OR (T ai Chi):ti,ab,kw

#39 #37 OR #38

#40 (Baduanjin):ti,ab,kw OR (Tuina):ti,ab,kw

#41 #14 OR #17 OR #20 OR #21 OR #24 OR #27 OR #30 OR #33 OR #36 OR #39 OR #40

#42 #11 AND #41

A total of 855 articles were retrieved. Trials 813, Reviews 41, Editorials 1, NE 855

was imported and the last 836 was removed.

2.2.7 Embase

Retrieval time: until March 1, 2021

#1 'Acupuncture'/exp **47,459**

#2 'Cupping Therapy'/exp **273**

#3 'Qigong'/exp **785**

#4 'Tai Chi'/exp **2,991**

#5 'Moxibustion':ab,ti OR 'Moxabustien':ab,ti OR 'Massage':ab,ti OR 'Zone Therapy':ab,ti OR 'Therapies, Zone':ab,ti OR 'Zone Therapies':ab,ti OR 'Therapy, Zone':ab,ti OR 'Massage Therapy':ab,ti OR 'Massage Therapies':ab,ti OR 'Therapies, Massage':ab,ti OR 'Therapy, Massage':ab,ti OR 'Cupping Therapy':ab,ti OR 'Cuppingtherapies':ab,ti OR 'Therapy,Cupping':ab,ti OR 'Cupping Treatment':ab,ti OR 'Cupping Treatments':ab,ti OR 'Treatment, Cupping':ab,ti OR 'Beguant':ab,ti OR 'Qigong':ab,ti OR 'Qi Gong':ab,ti OR 'Tai Ji':ab,ti OR 'Taichi':ab,ti OR 'Chi, Tai':ab,ti OR 'Tai Ji Quan':ab,ti OR 'Ji Quan,Tai':ab,ti OR 'Quan,Tai Ji':ab,ti OR 'Taiji':ab,ti OR 'Taijiquan':ab,ti OR 'Tlai Chi':ab,ti OR 'Tai Chi Chuan':ab,ti OR 'Baduanjin':ab,ti OR 'Tuina':ab,ti **19,301**

#6 'Complementarytherapies':ab,ti Or 'Therapies, Complementary':ab,ti Or 'Therapy, Complementary':ab,ti Or 'Complementary Medicine':ab,ti Or 'Medicine, Complementary':ab,ti Or 'Alternativemedicine':ab,ti Or 'Medicine,

Alternative':ab,ti Or 'Alternative Therapies':ab,ti Or 'Therapies,Alternative':ab,ti Or 'Therapy, Alternative':ab,ti **22,444**

#7 'Medicine, Traditional':ab,ti Or 'Home Remedies':ab,ti Or 'Home Remedy':ab,ti Or 'Remedies, Homeziy':ab,ti Or 'Remedy,Home':ab,ti Or 'Medicine, Primitive':ab,ti Or 'Primitivemedicine':ab,ti Or 'Medicine, Folk':ab,ti Or 'Folk Medicine;Medicine, Indigenous':ab,ti Or 'Indigenous Medicine':ab,ti Or 'Folk Remedies':ab,ti Or 'Folk Remedy':ab,ti Or 'Remedies, Folk':ab,ti Or 'Remedy, Folk':ab,ti Or 'Ethnomedicine':ab,ti **2,912**

#8 'Traditional Medicine'/exp104,319

#9 'Medicine, Herbal':ab,ti Or 'Hawaian Herbal Medicine':ab,ti Or 'Hawaiian Herbal Medicines':ab,ti Or 'Herbal Medicine, Hawaiian':ab,ti Or 'Herbal Medicines,Hawaiian':ab,ti Or 'Medicine, Hawaiian Herbal':ab,ti Or 'Medicines, Hawaiian-Herbal':ab,ti Or 'Laau Lapaau':ab,ti Or 'La Au Lapa Au':ab,ti Or 'Herbalism':ab,**ti** **388**

#10 'Herbal Medicine'/exp **22,834**

#11 'Traditional Chinese Medicine':ab,ti Or 'Chung Hsueh':ab,ti Or 'Hsueh, Chung':ab,ti Or 'Traditional Medicine, Chinese':ab,ti Or 'Zhong Yi Xue':ab,ti Or 'Chinese Traditionalmedicine':ab,ti Or 'Chinese Medicine, Traditional':ab,ti Or 'Tongue Diagnoses':ab,ti Or 'Traditional Tongue Diagnosis':ab,ti Or 'Traditional':ab,ti Or 'Traditional Tonguediagnoses':ab,ti Or 'Traditional Tongue Assessment':ab,ti Or 'Tongue Assessment, Traditional':ab,ti Or 'Traditional Tongue Assessments':ab,ti Or 'Medicine, Chinese Traditional':ab,ti **412,961**

#12 'Chinese Medicine'/exp **52,718**

#13 ('Vascular Neurocognitive Disorders':ab,ti Or 'Vascular Cognitive Disorders':ab,ti Or 'Vascular Neurocognitive Disorder':ab,ti Or 'Vascular Mild Cognitive Impairment':ab,ti Or 'Vascular Cognitive Impairment, No Dementia':ab,ti Or 'Post Stroke Cognitive Impairment':ab,ti Or 'Post-Stroke Dementia':ab,ti Or 'Subcortical Vascular Dementia':ab,ti Or 'Cognitive Impairment No Dementia Because Of Cerebrovasculardisease':ab,ti Or

'Ischemic Vascular Dementia':ab,ti Or 'Subcortical Vascular Disease':ab,ti Or 'Primary Degenerative Dementia':ab,ti) And 'Mixed Dementia':ab,ti Or 'Cerebrovascular Cognitive Impairment':ab,ti Or 'Multi Infarct Dementia':ab,ti Or 'Subcortical Ischemic Vascular Disease':ab,ti **1,166**

#14 'Arteriosclerotic Encephalopathies, Subcortical':ab,ti Or 'Encephalopathies, Subcortical Arteriosclerotic':ab,ti Or 'Subcortical Arteriosclerotic Encephalopathies':ab,ti Or 'Encephalopathy,Binswangers':ab,ti Or 'Encephalopathy, Binswanger':ab,ti Or 'Encephalopathy, Chronic Progressive Subcortical':ab,ti Or 'Subcortical Ischemie Vasculardementia':ab,ti **51**

#15 'Encephalopathy, Subcortical, Chronic Progressive':ab,ti Or 'Subcortical Encephalopathy, Chronic Progressive':ab,ti Or 'Subcortical Leukoencephalopathy':ab,ti Or 'Subcortical Arteriosclerotic Encephalopathy':ab,ti Or 'Arteriosclerotic Encephalopathy, Subcortical':ab,ti **178**

#16 'Binswangers Disease':ab,ti Or 'Subcortical Leukoencephalopathies':ab,ti Or 'Encephalopathy, Subcortical Arteriosclerotic':ab,ti **2**

#17 'Binswanger Disease':ab,ti Or 'Disease, Binswanger':ab,ti Or 'Chronic Progressive Subcortical Encephalopathy':ab,ti Or 'Binswanger Encephalopathy':ab,ti Or 'Leukoencephalopathy, Subcortical':ab,ti Or 'Leukoencephalopathies, Subcortical':ab,ti Or 'Vascular Dementias, Subcortical':ab,ti Or 'Arteriosclerotic Dementia':ab,ti Or 'Arteriosclerotic Dementias':ab,ti Or 'Dementia, Arteriosclerotic':ab,ti Or 'Dementias, Arteriosclerotic':ab,ti **169**

#18 'Acute Onset Vascular Dementia':ab,ti Or 'Subcortical Vascular Dementia':ab,ti Or 'Dementia, Subcortical Vascular':ab,ti Or 'Dementias, Subcortical Vascular':ab,ti Or 'Subcortical Vascular Dementias':ab,ti Or 'Vascular Dementia, Subcortical':ab,ti Or 'Dementias, Vascular':ab,ti Or 'Vascular Dementias':ab,ti Or 'Vascular Dementia':ab,ti Or 'Vascular Dementia, Acute Onset':ab,ti Or 'Lacunar Dementia':ab,ti Or 'Dementia, Lacunar':ab,ti Or 'Dementias, Lacunar':ab,ti Or 'Lacunar Dementias':ab,ti **9,485**

#19 'Dementias, Multiinfarct':ab,ti Or 'Multiinfarct Dementia':ab,ti Or 'Multiinfarct Dementias':ab,ti Or 'Multi-Infarct Dementia':ab,ti Or 'Multi Infarct Dementia':ab,ti Or 'Dementia Multi Infarct':ab,ti Or 'Dementia Multi-Infarcts':ab,ti Or 'Multi-Infarct,Dementia':ab,ti Or 'Multi-Infarcts, Dementia':ab,ti Or 'Dementia, Multiinfarct':ab,ti Or 'Dementia, Multi Infarct':ab,ti Or 'Dementias, Multi-Infarct':ab,ti Or 'Multi-Infarct Dementias':ab,ti Or 'Multiinfarct Dementia':ab,ti **1,144**

#20 'Multiinfarct Dementia'/exp **12,399**

#21 'Cognitive Dysfunction':ab,ti Or 'Cognitive Dysfunctions':ab,ti Or 'Dysfunction, Cognitive':ab,ti Or 'Dysfunctions, Cognitive':ab,ti Or 'Cognitive Impairments':ab,ti Or 'Cognitive Impairment':ab,ti Or 'Impairment, Cognitive':ab,ti Or 'Impairments, Cognitive':ab,ti Or 'Mild Cognitive Impairment':ab,ti Or 'Cognitive Impairment, Mild':ab,ti Or 'Cognitive Impairments, Mild':ab,ti Or 'Impairment, Mild Cognitive':ab,ti Or 'Impairments, Mild Cognitive':ab,ti Or 'Mild Cognitive Impairments':ab,ti Or 'Mild Neurocognitive Disorder':ab,ti Or 'Disorder, Mild Neurocognitive':ab,ti Or 'Disorders, Mild Neurocognitive':ab,ti Or 'Mild Neurocognitive Disorders':ab,ti Or 'Neurocognitive Disorder, Mild':ab,ti Or 'Neurocognitive Disorders, Mild':ab,ti Or 'Cognitive Decline':ab,ti Or 'Cognitive Declines':ab,ti Or 'Decline, Cognitive':ab,ti Or 'Declines, Cognitive':ab,ti Or 'Mental Deterioration':ab,ti Or 'Deterioration, Mental':ab,ti Or 'Deteriorations, Mental':ab,ti Or 'Mental Deteriorations':ab,ti **144,037**

#22 'Pharmacopunsture':ab,ti Or 'Acupuncture Treatment':ab,ti Or 'Acupuncture Treatments':ab,ti Or 'Treatment, Acupuncture':ab,ti Or 'Acupuncture':ab,ti Or'Pharmaceacupuncture Treatment':ab,ti Or 'Treatment, Pharmacoacupuncture':ab,ti Or 'Pharmacoacupuncture Therapy':ab,ti Or 'Therapy Pharmacacupunsture':ab,ti Or 'Asuretomies':ab,ti Or 'Acupunctures, Ear':ab,ti Or 'Ear Acupunctures':ab,ti Or 'Auricular Acupuncture':ab,ti Or 'Ear Acupuncture':ab,ti Or 'Acupuncture, Auricular':ab,ti Or 'Acupunctures,Auricular':ab,ti Or 'Auricular Acupunctures':ab,ti Or

'Acupuncture Point':ab,ti Or 'Point, Acupuncture':ab,ti Or 'Points, Acupuncture':ab,ti Or 'Acupoints':ab,ti Or 'Acupoint':ab,ti Or 'Analgesia, Acupuncture':ab,ti Or 'Acupunctureanesthesia':ab,ti Or 'Anesthesia, Acupuncturee':ab,ti **34,228**

#23 #1 OR #2 OR #3 OR #4 OR #5 OR #6 OR #7 OR #8 OR #9 OR #10 OR #11 OR #12 OR #22 **547,106**

#24 #13 OR #14 OR #15 OR #16 OR #17 OR #18 OR #19 OR #20 OR #21 **154,749**

#25 #23 AND #24 **3,216**

A total of 3216 articles were retrieved. NE 3216 is actually imported, and the last 3191 is removed.

2.2.8 ChiCTR and ClinicalTrials.gov

（1）ChiCTR

"Registration topic:" Vascular cognitive disorders "for retrieval, a total of 11 qualified studies, 6 studies have been concluded, but none of the published articles or uploaded data. Relevent information of eligible projects is shown in Table 2.

Table 2. Summary of Clinical Registration Research Retrieval of Traditional Chinese Medicine in the Treatment of Vascular Cognitive Disorder (ChiCTR)

Serial number	Registration number	Registered subject	Registered unit	Research type	Registration time	Whether the research results are published or uploaded
1	ChiCTR-IIR-17011513	A randomized controlled study on the treatment of vascular cognitive impairment without dementia by acupuncture with the method of regulating supervision and tranquilizing the mind	Shanghai Hospital of Traditional Chinese Medicine	Intervention studies	2017-05-27	NO

2	ChiCTR1900025713	Systematic Study on Dialectical Criteria of "Kidney-Yang Deficiency Syndrome" of Vascular Cognitive Disorder	Dongfang Hospital of Beijing University of Chinese Medicine	Intervention studies	2019-09-06	Not concluding
3	ChiCTR1800017398	To investigate the functional imaging study of electroacupuncture "Baihui" and "Shenting" in the intervention of non-dementia vascular cognitive impairment based on brain default mode network	Fujian University of Traditional Chinese Medicine	Intervention studies	2018-07-28	NO
4	ChiCTR2000030286	To explore the mechanism of brain effect of head acupuncture in the treatment of vascular dementia based on DTI	Shuguang Hospital Affiliated to Shanghai University of Chinese Medicine	Intervention studies	2020-02-27	Not concluding
5	ChiCTR1900023170	The clinical efficacy of Hengqing I prescription in the treatment of vascular dementia was observed in a multicenter randomized double-blind placebo-controlled study	Shanghai Sixth People's Hospital	Intervention studies	2019-05-15	Not concluding
6	ChiCTR1900020637	Analysis of Urine Specific Metabolites in Patients with	Shanghai Sixth People's Hospital	Intervention studies	2019-01-11	Not concluding

		Syndrome of Kidney Deficiency and Phlegm Stasis in Vascular Dementia and Study on the Intervention of Hengqing I Recipt				
7	ChiCTR1800017489	Study on the effect of zhisanzhen on vascular dementia	The First Affiliated Hospital of Guangzhou University of Chinese Medicine	Intervention studies	2018/08/01	NO
8	ChiCTR1800017359	Evaluation of Shenma Yizhi Decoction on the Improvement of Neurological Defect in Patients with Vascular Dementia (Deficiency of Blood Stasis and Lian-Yang Syndrome)	Xiyuan Hospital, China Academy of Chinese Medical Sciences	Intervention studies	2018/07/25	NO
9	ChiCTR-ONRC-08000277	Brain functional imaging study of different acupoint effects in vascular dementia patients with acupuncture intervention	The First Hospital of School of Traditional Chinese Medicine, Southern Medical University	Observational study	2008/12/31	NO
10	ChiCTR-TRC-14004613	Clinical efficacy and safety of Ceruotong capsule in the treatment of vascular dementia: a randomized, double-blind, placebo-parallel	Xuanwu Hospital of Capital Medical University	Intervention studies	2014/01/09	NO

		controlled, multicenter II phase III clinical trial and an open phase continuation trial				
11	ChiCTR-TRC-13003916	A randomized, double-blind, placebo-parallel controlled, multicenter II phase II clinical trial of the efficacy and safety of Ceruotong capsule in the treatment of vascular dementia	Xuanwu Hospital of Capital Medical University	Intervention studies	2013/04/07	NO

（2）ClinicalTrials.gov

Of the 11 traditional Chinese medicine studies with vascular cognitive impairment that met the requirements, one had published articles, which are indicated in the table below. Relevent information of eligible projects is shown in Table 3.

Table 3 Summary of Clinical Registration Research Retrieval for the Treatment of Vascular Cognitive Disorder with Traditional Chinese Medicine (ClinicalTrails. Gov)

Serial number	Registration number	Registered subject	Research type	Registration time	Whether the research results are published or uploaded
1	NCT04087499	Potential Role of Acupuncture Treatment in Neuronal and Network Dysfunction in Patients With Vascular Cognitive Impairment	Intervention studies	2019.9.12	Not concluding
2	NCT04301466	Efficacy and Safety of Qi Zhi Tong Luo Capsule in Vascular Cognitive Impairment	Intervention studies	2020.3.10	Not concluding
3	NCT02752867	An fMRI Study of Jianpi Yishen Huatan Granules for Cognitive Impairment After Acute Cerebral Infarction	Intervention studies	2016.4.27	NO

4	NCT02641886	The Study of Jian Pi Yi Shen Hua Tan Granules in Cognitive Impairment After Acute Cerebral Infarction	Intervention studies	2015.12.30	NO
5	NCT01475578	Study of STA-1 Capsule in Patients With Vascular Dementia (Marrow-Sea Deficiency)	Intervention studies	2011.11.11	NO
6	NCT03230071	Efficacy and Safety of TMBCZG in Mild to Moderate Vascular Dementia	Intervention studies	2017.7.26	NO
7	NCT02453932	Efficacy and Safety of Tianzhi Granule in Mild to Moderate Vascular Dementia	Intervention studies	2015.5.27	NO
8	NCT01761227	Efficacy and Safety of Fufangdanshen Tablets in Mild to Moderate Vascular Dementia	Intervention studies	2013.1.4	NO
9	NCT03789760	The Clinical Trial of Chinese Herbal Medicine (SaiLuoTong) Capsule	Intervention studies	2018.12.30	Not concluding
10	NCT01978730	The Clinical Trial of Chinese Herbal Medicine SaiLuoTong Capsule	Intervention studies	2013.11.7	Jia, J., et al., Efficacy and safety of the compound Chinese medicine SaiLuoTong in vascular dementia: A randomized clinical trial. Alzheimer's and Dementia: Translational Research and Clinical Interventions, 2018. 4: p. 108-117. Included in the database.
11	NCT04498962	The Effect of Danzhu Fuyuan Granule as Adjunctive Therapy for Chronic Stable	Intervention studies	2020.8.5	Not concluding

3.Literature screening and classification

Four Chinese databases, three English databases and two clinical trial registries imported a total of 57821 titles from NE literature management software. A total of 10473 duplicates were found in the literature review, and a total of 47,347 literatures in both Chinese and English were obtained after deconstructing.

3.1 Preliminary screening:

Through reading titles and abstracts, 39,375 unrelated literatures were prelimi narily screened out, and the remaining 7,972 literatures were related to traditio nal Chinese medicine. Among them, there were **3288 randomized controls**;219 non-randomized controls;109 systematic reviews and systematic evaluation reev aluation;30 meta-analyses;6 clinical guidelines, consensus and pathways; A coho rt study of 43;116 cross-sectional studies;83 case controls; Case series 343;25 c ase reports;11 papers of scheme registration;967 articles were reviewed.174 fam ous experience;2521 animal and cell experiments; It is inconvenient to classify 37 articles.

3.2 Literature screening of GbE-related RCTs:

In the 3288RCTs introduced into NE, 100 RCTs related to GbE were retriev ed through the keywords of Ginkgo leaf, Ginkgo leaf, Ginkgo biloba, GbE, etc. Among which 32 were inconsistent with the study intervention measures, 42 were excluded after reading the full text, 3 were repeated, and 23 were finally included.
